# Application of pulse-modulated radio-frequency atmospheric pressure glow discharge for degradation of doxycycline from a flowing liquid solution

**DOI:** 10.1038/s41598-022-11088-w

**Published:** 2022-05-05

**Authors:** Anna Dzimitrowicz, Magda Caban, Dominik Terefinko, Pawel Pohl, Piotr Jamroz, Weronika Babinska, Piotr Cyganowski, Piotr Stepnowski, Ewa Lojkowska, Wojciech Sledz, Agata Motyka-Pomagruk

**Affiliations:** 1 Department of Analytical Chemistry and Chemical Metallurgy, Wroclaw University of Science and Technology, 27 Wybrzeze St. Wyspianskiego, 50-370 Wroclaw, Poland; 2 Department of Environmental Analysis, Faculty of Chemistry, University of Gdansk, 63 Wita Stwosza, 80-308 Gdansk, Poland; 3 Laboratory of Plant Protection and Biotechnology, Intercollegiate Faculty of Biotechnology University of Gdansk and Medical University of Gdansk, University of Gdansk, 58 Abrahama, 80-307 Gdansk, Poland; 4 Department of Polymer and Carbonaceous Materials, Wroclaw University of Science and Technology, 27 Wybrzeze St. Wyspianskiego, 50-370 Wroclaw, Poland

**Keywords:** Environmental chemistry, Environmental chemistry, Pollution remediation, Water microbiology

## Abstract

Doxycycline (DOX), an antibiotic commonly used in medicine and veterinary, is frequently detected in natural waterways. Exposition of bacteria to DOX residuals poses a selective pressure leading to a common occurrence of DOX-resistance genetic determinants among microorganisms, including virulent human pathogens. In view of diminishment of the available therapeutic options, we developed a continuous-flow reaction-discharge system generating pulse-modulated radio-frequency atmospheric pressure glow discharge (pm-rf-APGD) intended for DOX removal from liquid solutions. A Design of Experiment and a Response Surface Methodology were implemented in the optimisation procedure. The removal efficiency of DOX equalling 79 ± 4.5% and the resultant degradation products were identified by High-Performance Liquid Chromatography–Diode Array Detection, Liquid Chromatography Quadruple Time of Flight Mass Spectrometry, Ultraperformance Liquid Chromatography–Tandem Mass Spectrometry, total organic carbon, total nitrogen, Attenuated Total Reflectance Furrier Transform–Infrared, and UV/Vis-based methods. The pm-rf-APGD-treated DOX solution due to the generated Reactive Oxygen and Nitrogen Species either lost its antimicrobial properties towards *Escherichia coli* ATCC25922 or significantly decreased biocidal activities by 37% and 29% in relation to *Staphylococcus haemolyticus* ATCC29970 and *Staphylococcus aureus* ATCC25904, respectively. Future implementation of this efficient and eco-friendly antibiotic-degradation technology into wastewater purification systems is predicted.

## Introduction

Increasing antibiotic resistance among virulent human pathogens is nowadays one of the most alarming medical threats. It results from the fact that these drugs are often wrongly prescribed or administered in inadequate doses. Antibiotics also find applications for preventive non-therapeutic purposes or boosting animal growth in many countries. For instance in the USA, 50% of 22,700 tons of these substances produced each year is utilized in animal husbandry, agriculture, and aquafarming^1^. In consequence of high production and application rates, these compounds, either in indigenous or metabolized forms, may reach surface waters, groundwater or sediments together with washes offs from the fields, as well as direct discharges from animal farming/food processing and only partially purified municipal, medical or pharmaceutical effluents^2^. It needs to be taken into consideration that common sewage treatment plants are inefficient in decomposition of this group of drugs. Due to potent biocidal activities of these substances, the antibiotics-triggered changes in the population of bacteria present in the wastewater treatment system might impede the effectiveness of organic matter degradation processes. When semi- or non-degraded antibiotics reach natural environment, in spite of disturbances in the nitrification reaction^3^, adverse effects towards early developmental stages of aquatic organisms^4^, disturbances in the biodiversity of microbial communities^5^ and acquiring resistance to these molecules^6^ are all to be suspected to take place. Subjection to the selective pressure of sub-lethal doses of antibiotics, leads to selection of natural bacterial populations harbouring resistance-associated genetic determinants. These genes or gene clusters are transmitted either vertically or horizontally, while the latter approach involves plasmids, mobile genetic elements or bacteriophages^7^. Acquisition of these genetic cassettes may also occur via engulfment of free DNA^7^. Therefore, antibiotic resistance, which is commonly detected in microorganisms of waterways or soil origin^6^, is transferred and accumulates in resistant or multi-drug resistant human pathogens such as *Staphylococcus aureus*, *Klebsiella pneumoniae*, *Pseudomonas aeruginosa*, *Streptococcus pneumoniae*, *Salmonella* sp. *Escherichia coli*, *Acinetobacter baumanii*, *Enterocccus* sp., *Clostridium difficile* etc^8^. For that reason, antibiotics, including doxycycline (α-6-deoxy oxytetracycline; DOX), are regarded as emerging pollutants that bring new challenges in terms of their efficient removal. Due to the lack of the associated water quality regulations, the presence of these substances certainly poses a serious risk to the natural environment and human health^9^.

In this study, we focused on DOX that is an inexpensive, semi-synthetic antibiotic belonging to the class of second-generation tetracyclines. This broad spectrum drug of bacteriostatic properties is used for over 50 years^10^ to inhibit the growth of gram-positive and gram-negative bacteria, including both aerobic and anaerobic ones. In more detail, DOX interferes with protein synthesis on 70 S bacterial ribosomes via prevention of the attachment of aminoacyl-tRNA to the acceptor site on the mRNA-translating complex. From a clinical point of view, it is worth to consider its rather good tolerance, wide therapeutic–toxic window, fast intestinal absorption, great fluid and tissue penetration, and prolonged half-life in blood^11,12^. DOX is widely used in medical practice for treatment of many ailments and diseases, e.g. genitourinary, gastrointestinal or respiratory tract infections, acne, rosacea, Lyme disease, cholera, sepsis or in prevention of malaria^9–11^. In spite of the fact that DOX is frequently prescribed for combating bacterial infections in humans, it also finds applications in veterinary medicine or as a disease-preventing or growth-boosting additive to animal feed^13^. Unfortunately, DOX is not metabolized to a great extent after oral administration because approx. 40–45% of the ingested dose is released in urine post 72 h^11^. Studies on the persistence of this drug revealed that the half-life of DOX in, for instance, pig manure aged under field conditions equals 25.7 days^14^. In terms of the aquatic environment, Zaranyika et al.^9^ showed that the concentration of DOX introduced into river water samples dropped from 1 µg mL^−1^ to over 0.1 µg mL^−1^ during 90-days incubation. Therefore, beside frequent applications, the endurance of this drug in the natural aquatic environment might be the reason why notable concentrations of DOX, reaching 17.9 ng L^−1^ or 0.95 ng L^−1^ have been detected in the rivers of USA or China, respectively, in particular in a close distance from large urban areas, feedlots or fish ponds^9^. Even after exposition to low concentrations of DOX, the following resistance mechanisms are triggered in bacterial populations: changes in the site of action of the drug, a diminished uptake or pumping-out of the active molecule via efflux pumps^11,12^.

In order to address environmental and health-related concerns associated with acquiring of antibiotic resistance determinants to animal and human bacterial pathogens, several various approaches designated for degradation of these drugs have been proposed to the present day. By now, hydrothermal processes, adsorption strategies as well as conventional and electrochemical advanced oxidation processes have been investigated aiming for decomposition of antibiotics^15^. Although these processes are well-studied and employed in some wastewater purification systems, total degradation of the introduced organic compounds is regarded to be time-consuming with these techniques^16^. Additionally, notable toxicity towards aquatic organisms of the resultant intermediates and occasionally not satisfactory decay rates of the studied compounds have been reported^4,16^. A very promising and more effective alternative to degradation of antibiotics than the above-listed techniques might be the use of cold atmospheric pressure plasma (CAPP)^17^. The possible application of CAPPs for potent decomposition of organic compounds, including drugs, is associated with production of different reactive species, including reactive oxygen species (ROS), reactive nitrogen species (RNS), and solvated electrons (e^−^_aq_), in addition to UV radiation and electromagnetic field that accompany the CAPP operation^18^. Because the first mentioned reactive individuals exhibit defined red-ox potentials, the CAPP-based technology and processes might be useful for wastewater treatment facilities.

Up to now, several CAPP-based technologies have been applied for degradation of various classes of antibiotics e.g. fluoroquinolones^16,17,19^, tetracyclines^17,20,21^ or sulfonamides^17^. Although different CAPP systems have been investigated up to now for decomposition of several antibiotics belonging to diverse groups^17^, to the best of our knowledge, there are only two studies reporting CAPP-based degradation of DOX from aqueous solutions^20,21^. The impact of two types of CAPPs treatments, employing either gas-phase pulsed corona discharge^21^ or dielectric barrier discharge above or below water^20^, on the DOX degradation efficacy has been briefly discussed in these papers. Unfortunately, the effect of operating parameters on the DOX decomposition efficiency in liquid disposals has not been examined in these works^20,21^. Additionally, all of the before-developed CAPP-based reaction-discharge systems intended for DOX degradation, work in non-flowing modes, which means that the purified volume of the wastewater containing DOX is limited by the total volume of the water container^20,21^.

Here, we have developed and optimized a novel CAPP-based continuous-flow reaction-discharge system, employing pulse-modulated radio-frequency atmospheric pressure glow discharge (pm-rf-APGD), for effective degradation of DOX. Moreover, we have proven either a loss or a substantial decrease in biocidal properties of the studied drug. The basic research hypothesis assumed that production of ROS, RNS, and e^−^_aq_, originating from pm-rf-APGD operation in contact with a flowing (drug-containing) effluent, would lead to highly effective degradation of DOX under initially defined and verified optimal operating of this CAPP system. In order to find these optimal operating parameters, the advanced statistical method, i.e. design of experiments (DoE) followed by response surface methodology (RSM), was applied. The efficiency of DOX degradation was evaluated using a high-performance liquid chromatography—diode array detection (HPLC–DAD) system. The products of DOX degradation, resulting from the pm-rf-APGD treatment, have been revealed by using ultraperformance liquid chromatography–tandem mass spectrometry (UPLC–MS/MS) and liquid chromatography-quadrupole time of flight mass spectrometry (LC-QToF MS). Additionally, to state the impact of the studied pm-rf-APGD system on the chemical composition of the DOX-containing effluent, its total organic carbon (TOC) and total nitrogen (TN) contents were determined in addition to  conducting qualitative analysis by attenuated total reflectance furrier transform-infrared (ATR FT-IR) spectroscopy and UV/Vis absorption spectrophotometry. Afterwards, changes in the antimicrobial properties of the pm-rf-APGD-treated DOX solution in contrast to the control solution were assessed towards important from a clinical point of view human opportunistic pathogens i.e. *E. coli*, *S. aureus,* and *Staphylococcus haemolyticus*. Finally, pm-rf-APGD-liquid reactions and processes, possibly leading to degradation of DOX, were proposed and discussed in connection with results of measurements of selected ROS and RNS by colorimetric methods.

## Methods

### Working solutions preparation

A 1000 µg mL^−1^ DOX stock solution was prepared by dissolving 57.7 mg of doxycycline hyclate (A&A Biotechnology, Poland) in 50 mL of de-ionized water. Then, 3 working solutions for the optimization procedure, including 10.0, 55.0 and 100 µg mL^−1^, as well as working solutions for the validation procedure, were prepared by properly diluting the stock standard solution. In addition, 8 working standard solutions for the calibration of the HPLC–DAD system were prepared and ranged from 0.002 to 50.0 µg mL^−1^. All above-mentioned solutions were stored in brown vials at 4 °C.

### Defining optimal operating conditions for DOX degradation by optimization of the flow-through pm-rf-APGD-based reaction-discharge system

For DOX degradation, a novel, flow-through pm-rf-APGD-based reaction-discharge system, allowing for stable ignition and operation of the CAPP source under defined operating conditions, was used (Fig. S1, Table S1). In the studied system, introduction of DOX-containing solutions occurred through a quartz capillary (OD = 4.00 mm). On this capillary, a graphite tube (OD = 6.00 mm) was mounted. By attaching a Pt wire to the graphite tube and covering it with a current resistant tape, electrical contact was provided to the continuously pumped solution (flowing liquid electrode, FLE). The second electrode was a sharpened, pin-type metallic rod (OD = 3.2 mm), coaxial with the quartz tube, and placed about 3.0 mm away from the edge of this tube. pm-rf-APGD was generated in the gap between the surface of the FLE and the latter metallic electrode. A high-voltage (HV) potential was provided to both electrodes by using a Dora pm-rf alternating current supply (Dora Electronic Equipment, Poland). pm-rf-APGD was initialized by applying a (50 kHz) voltage wave, modulated at frequencies within 500–1400 Hz, with a duty cycle in the range from 30 to 50% (Table S1). After introducing the DOX solution to the pm-rf-APGD-based reaction-discharge system, portions of this solution were gathered into brown vials for further analyses.

To establish the operating parameters of the pm-rf-APGD-based reaction-discharge system that would be suitable for achieving efficient decomposition of DOX dissolved in the continuously introduced solution with the use of the studied CAPP source, a multiparameter optimization based on DoE was applied. In order to set the optimization experiment, a Box-Behnken response surface design was utilized (Table S1). Here, 15 randomized treatments were included according to the matrix involving the information on a standard run, a run order of the experiments, and conditions related to 3 different levels of the studied operating parameters (uncoded and codded (− 1, 0, + 1) in brackets). The above-mentioned operating parameters were as follows: the DOX concentration (A, in µg mL^−1^), the flow rate of the FLE solution (B, in mL min^−1^), and the duty cycle for radio-frequency pulses (C, %) modulating the alternating current that supplies the electrodes of the pm-rf-APGD system. The traced response variables for the undertaken Box-Behnken response surface design were pH of the DOX-containing solution measured after the pm-rf-APGD treatment and temperature (T, °C). In the case of the pm-rf-APGD system, the duty cycle was directly related to the discharge power at a given voltage and frequency of the rf altering current. The latter parameters were not changed; hence, the forwarded power to the pm-rf-APGD system was only related to the duty cycle. Here, the highest duty cycle and the highest discharge power were applied. For the applied duty cycle such as 30, 40 and 50%, the total discharge power was found to be: 51, 68 and 84 Watts, respectively. To assess the precision of the response variables of the applied Box-Behnken response surface design, 3 center points were included in the matrix (Table 1). Considering values of traced responses at these center points, the precision was high, i.e. 3.2% for pH (with a mean of 3.05) and 5.1% for T (with a mean of 34.3 °C). All treatments for the Box–Behnken response surface design were carried out in one experimental block. Accordingly, DOX-containing solutions (at 3 different concentrations; Table S1) were continuously pumped into the pm-rf-APGD reaction-discharge system (at 3 different flow rates; Table S1), which was operated under the pulsed modulated radio-frequency (pm-rf) alternating current with a duty cycle at 3 different levels (Table S1). Due to the contact of the solution surface with the discharge phase and plasma-liquid interactions taking place in the interfacial zone, short- and long-term reactive oxygen and nitrogen species (RONS) were produced leading to decomposition of DOX in the introduced solutions. The portions of the overflowing solutions were collected after the pm-rf-APGD treatment and their T and pH were immediately measured. To do so a Hanna Instruments thermometer (Poland) and an Elmetron CPC-505 pH-meter (Poland) were applied.

**Table 1:** The analysis of variance (ANOVA) table for the response surface model developed to describe the effect of operating parameters of pulse-modulated radio-frequency atmospheric pressure glow discharge (pm-rf-APGD), i.e. A-DOX concentration in the flowing liquid electrode (FLE) solution (in µg mL^−1^), B-FLE solution flow rate (in mL min^−1^), and C-duty cycle for the pulse modulated radio-frequency alternating current, on temperature (T) of DOX-containing solutions treated by pm-rf-APGD.

Source	*DF*	Adj SS	Adj MS	*F*-value	*P*-value
Model	3	42.823	14.274	6.74	0.009 < 0.1
Linear	2	19.163	9.582	4.52	0.040 < 0.1
A	1	1.700	1.700	0.80	0.391 > 0.1
B	1	18.816	18.816	8.88	0.014 < 0.1
Squared	1	28.958	28.958	13.67	0.004 < 0.1
B^2^	1	28.958	28.958	13,67	0.004 < 0.1
Error	10	21.191	2.119		
Lack-of-fit	8	19.971	2.496	4.09	0.211 > 0.1
Pure error	2	1.220	0.610		
Total	13	64.014			
Regression equation and model summary					
T (°C) = 0.710 – 1.80 × 10^+1^ × B + 1.68 × 10^−1^ × C – 2.91 × B^2^					
S = 1.456, R^2^ = 66.9%, R^2^-adjusted = 57.0%					

DF: Degrees of freedom. Adj SS: adjusted sums of squares. Adj MS: adjusted mean squares. *F*-value: the value of the Fisher–Snedecor test. *P*-value: probability value.

### Design of experiments with response surface methodology

The collected data (values of the response variables) for the applied response surface design were analyzed by using full quadratic functions, including the effect of linear (A, B, C) and squared (A^2^, B^2^, C^2^) terms as well as two-way interaction terms (A × B, A × C, B × C). Equations of these functions used to model the measurement data for each of the tested responses, i.e. T and pH, were as follows: d_0_ + a_1_A + b_1_B + c_1_C + a_2_A^2^ + b_2_B^2^ + c_2_C^2^ + a_3_A × B + b_3_A × C + c_3_B × C, where d_0_, a_1_–a_3_, b_1_–b_3_ and c_1_–c_3_ corresponded to regression coefficients. To find statistically significant terms in the response surface regression models for T and pH, the backward elimination of terms algorithm was used at the 90% significance level (α to remove equal to 0.1), requiring a hierarchical model at each step of the algorithm and for all terms. To assess the fit of the response surface regression models, the analysis of variance (ANOVA) test was used, enabling to determine whether the proposed relationship between the response variables and the set of terms is statistically reliable and will be proven useful to explain the response at the given operating conditions. Accordingly, the goodness-of-fit of the response surface regression models was indicated by the coefficient of determination (R^2^) and the adjusted R^2^. *p*-values determined for the response surface regression models with the included terms are shown in Table 1 (for T) and Table 2 (for pH) to present the statistical significance of these models. Residuals between the measured data and the data fitted by the developed response surface regression models were also visually inspected basing on the appropriate normal probability plots (a possibility to check if the residuals are normally distributed or not) and scatter plots of the standardized residuals versus the run order (a possibility to check whether the residuals are independent). The Box-Behnken response surface design experiments were also carried out considering the degradation efficiency of the target compound, determined by the HPLC–DAD method, as the response of the system under different conditions of A, B and C parameters. In this case, a similar approach was used as before, i.e., the backward elimination algorithm was applied at α = 0.1. The model assessed was also established to be valid, i.e., the *p*-value for the model was 0.004 (<< α) and the *p*-value for the lack-of-fit test was 0.198 (> α). The goodness-of-fit was indicated by the quite high R^2^ and adjusted R^2^ value (96.3 and 89.7%, respectively). The equation of the response surface was as follows: Degradation efficiency, % = 124.0 + 0.136 A − 1.44 B − 1.398 C − 0.000231 A^2^ + 0.146 B^2^ + 0.01823 C^2^ − 0.0456 AB − 0.00278 AC + 0.0133 BC.

**Table 2:** The analysis of variance (ANOVA) table for the response surface model developed to describe the effect of the operating parameters of the pulsed radio-frequency atmospheric pressure glow discharge (pm-rf-APGD), i.e., A-DOX concentration in the flowing liquid electrode (FLE) solution (in µg mL^−1^), B-FLE solution flow rate (in mL min^−1^), and C-duty cycle for the pulse-modulated radio-frequency alternating current, on pH of DOX-containing solutions treated by pm-rf- APGD.

Source	*DF*	Adj SS	Adj MS	*F*-value	*P*-value
Model	4	0.07919	0.01980	4.28	0.028 < 0.1
Linear	3	0.03732	0.01244	2.69	0.103 > 0.1
A	1	0.00211	0.00211	0.46	0.515 > 0.1
B	1	0.01901	0.01901	4.11	0.070 < 0.1
C	1	0.01620	0.01620	3.50	0.091 < 0.1
Squared	1	0.04186	0.04186	9.04	0.013 < 0.1
A^2^	1	0.04186	0.04186	9.04	0.013 < 0.1
Error	10	0.04630	0.00463		
Lack-of-fit	8	0.04024	0.00503	1.66	0.430 > 0.1
Pure error	2	0.00607	0.00303		
Total	14	0.12549			
Regression equation and model summary					
pH = 3.235 – 5.39 × 10^−3^ × A + 4.87 × 10^−2^ × B – 4.50 × 10^−3^ × C + 5.20 × 10^−5^ × A^2^					
S = 0.068, R^2^ = 63.1%, R^2^-adjusted = 48.3%					

DF: Degrees of freedom. Adj SS: adjusted sums of squares. Adj MS: adjusted mean squares. *F*-value: the value of the Fisher–Snedecor test. *P*-value: probability value.

At the end, to find the most favourable working conditions of the flow-through pm-rf-APGD-based reaction-discharge system for decomposition of DOX dissolved in aqueous solutions, optimal operating parameters settings were selected considering individual desirability functions for T and pH, i.e. dT and dpH, and the value of the composite desirability function, i.e. D = (dT × dpH)^1/2^. It was specified that the most favourable conditions for decomposition of the above-mentioned antibiotic would be achieved if T of the solution after its pm-rf-APGD treatment would reach the highest value (dT = 1) while its pH would be the lowest (dpH = 1). Under these operating conditions, it could be expected that the concentration of different RNS and ROS, including long-term H_2_O_2_, O_3_, NO_3_^−^, as well as short-term like ·OH, NO_2_^−^, O_2_^−^, and O_2_^1^ (singlet oxygen), would be the highest and should be involved in the DOX degradation process. The high T of the solution would enhance the constant rates of decay/degradation reactions for this antibiotic. For this reason, i.e. in order to identify RNS and ROS produced in the gaseous phase of the operated pm-rf-APGD system, optical emission spectrometry (OES) was employed. The OES measurements were conducted as follows: the emitted radiation of pm-rf-APGD was imaged by means of a UV achromatic lens (f = 60) on the entrance slit (10 µm) of a Shamrock SR-500i spectrograph (Andor, UK). The spectrograph was equipped with 1200 (range 300–900 nm) and 1800 (range 200–400 nm) grooves mm^−1^ holographic gratings and a UV/Vis CCD Newton DU-920P-OE camera (Andor, UK). A full vertical biding (FVB) mode of the CCD camera and an integration time of 0.1 s were applied in each case. The Solid S software (Andor UK) was utilized for data imaging and processing.

Finally, the validation procedure was performed in order to check the accuracy of the established statistical models. In this case, the pm-rf-APGD-based reaction-discharge system was operated under the following operating conditions selected on the basis of both models (T and pH), i.e. the FLE solution flow rate: 2.8 mL min^−1^, the duty cycle for the pulse-modulated radio-frequency alternating current of 50%, and the DOX concentration in the solution delivered to the pm-rf-APGD-based reaction-discharge system: 51.5 µg mL^−1^. Considering the maximal degradation efficiency, the optimal settings of the parameters of the pm-rf-APGD system found and based on this model were as follows: A = 10 µg mL^−1^, B = 2.0 mL min^−1^ and C = 50%. These parameters settings resulted in the degradation efficiency of 97.8 ± 8.1%. The particular significance needs to be attributed to the concentration of DOX because the setting of this parameter has to lead to the experimentally observable bacterial growth inhibition zones in the non-plasma treated doxycycline-containing solution. In contrast to the plasma-degraded antibiotic, the concentration of the biologically active compound is required to be high enough, i.e. equal at least 50 mg L^−1^, to limit the growth of the pathogenic microorganisms. The 10 µg mL^−1^ concentration of DOX yielded by the model could not be utilized in the experimental part of this study as it was not sufficient to inhibit the growth of the studied microorganisms. In the case of two latter parameters, i.e., B and C that are directly related to the operation of the flow-through CAPP system applied, their optimal settings corresponded to the optimal values found based on the models assessed using T (temperature) and pH of the pm-rf-APGD-treated solutions. In particular, the newly assessed model confirmed that the discharge power should be the highest, as obtained by using the 50% duty cycle. What is more, when the optimal settings found using the models for T and pH were applied, i.e., A = 51.5 µg mL^−1^, B = 2.8 mL min^−1^ and C = 50%, the model established for the degradation efficiency resulted in the response of 91.3 ± 4.3%, which was close to the value assessed at the validation experiment of the first two models, i.e., 79 ± 4.5%. In this case, it was possible to perform the planned microbiological assays. All these figures show that the optimal settings found based on the model assessed for the degradation efficiency and, particularly those related to the operation of the flow-through pm-rf-APGD system, are as good as the optimal settings established applying the models for T and pH. In this way, the assumptions about the production of RONS and the degradation efficiency at the highest T and the lowest pH of the pm-rf-APGD treated solution were reasonably valid. After subjecting the DOX-containing solutions to the system, the CAPP-treated solution was collected and its T and pH were measured to compare with the values established by both models.

### Estimation of the DOX removal efficiency under the optimal operating conditions of pm-rf-APGD

To find out the impact of the pm-rf-APGD treatment on the removal efficiency of DOX from aqueous solutions, the HPLC–DAD analysis was conducted. The prepared standard working solutions (see the chapter *Working Solution Preparation*) were stable at least 1 month at 4 °C. For HPLC–DAD measurements, a Nexera XR (Shimadzu) system was used and it was composed of: an autosampler SIL-20AC, a pump LC-20AD, a column oven CTO-20AC, and a detector SPD-M20A. The column used was a Gemini Gemini-NX (C18, 5 µm, 110A, 150 × 4.6 mm). The column oven was set to 27 °C. The autosampler oven temperature was 10 °C. The injection volume was 25 µL. The mobile phase was 2-component and included the component A (0.1% formic acid, pH 2.6) and the component B (acetonitrile, ACN). The gradient elution was used and looked as follows: 0 min – 15% B, 10 min – 50% B, 12 min – 50% B, 15 min – 15% B. The flow rate of the mobile phase was 1 mL/min. The total time of each chromatographic run was 15 min. The retention time of DOX was 5.6 min. The detector was set to measure the wavelengths from 190 to 800 nm, while quantification of DOX was performed at 348 nm. The linearity of the detector response was verified to be between 0.002 and 50.0 µg mL^−1^. The limit of quantification (LOQ) was assessed to be 0.004 µg mL^−1^, while the limit of detection (LOD) was LOQ/3. The regression coefficient of the calibration curve was 0.9998. The precision as determined by the relative standard deviation (RSD) was in the range of 1.08–5.80% (at least 3 repetitions of the injection for each of 8 concentration levels).

### Identification of the DOX degradation products following pm-rf-APGD treatment under the optimal operating conditions

To reveal the DOX degradation products, the UPLC-MS/MS analyses were conducted with a UPLC-MS/MS instrument (Agilent Technologies) that consisted of a liquid chromatograph 1290 Infinity and a mass spectrometer 6550 iFunnel Q-TOF. The column ZORBAX Eclipse Plus C18 (Rapid Resolution HD 2.1 × 50 mm, 1.8 µm, Agilent) was used. The mobile phase was 2-component and included the component A (0.1% formic acid in water with 3% ACN) and the component B (0.1% formic acid in ACN). The flow rate of the mobile phase was 400 µL min^−1^. The gradient program for elution was set as follows: from 2% of B to 100% of B in 7 min. Recording the full mass spectra, the mass range of 100–1000 was monitored in negative and positive ions modes. The instrument was tuned before the analysis and the following optimized conditions were set: the capillary voltage of 3.5 kV, the gas temperature of 250 °C, the sheath gas temperature of 300 °C, the sheath gas flow rate of 11 L min^−1^, the collision gas flow rate of 0.2 mL min^−1^, the fragmentor voltage of 175 V, and the nozzle voltage of 2 kV. The injection volume was 1 µL. The analyzed samples were thawed, vortex mixed and filtrated (0.2 µm pore size PET filters), and their 1-mL portions were transferred to brown-glass chromatographic vials prior to measurements.

### Impact of pm-rf-APGD used under the optimal operating conditions on the chemical structure of DOX

To assess the effect of pm-rf-APGD on DOX under optimal operating parameters of the applied flow-through CAPP system, the TOC, TN, ATR FT-IR, and UV/Vis analyses of the DOX-containing solution subjected to the CAPP treatment were performed. In addition, the untreated DOX-containing solution was analyzed for comparison. The concentrations of TOC and TN were determined by employing a Multi N/C 3100 Analytik Jena (Jena, Germany) instrument. For determination of TOC in the analyzed samples, the non-purgeable organic carbon (NPOC) method was applied. The content of TN in the analyzed samples was assessed in terms of nitrate-nitrogen (NO_3_–N), nitrite-nitrogen (NO_2_–N), ammonia–nitrogen (NH_3_–N), and organically bonded nitrogen. The TOC/TN measurements were carried out in brown vials to protect DOX from eventual uncontrolled degradation under the Vis light. The TOC/TN analyses were conducted by pouring brown vials with 10 mL of the analyzed samples and acidifying them with 500 µL of 2 mol L^−1^ HCl (Avantor Performed Materials, Poland) to reach pH 2 (HCl was added to remove CO_2_). Additionally, the content of TOC/TN in de-ionized water was measured as a control. The collected data were statistically tested with the aid of GraphPad Prism 8.0, by using the unpaired *t* test (Tukey’s post-hoc test, parametric analysis) and obtaining two-tailed *p* values.

The ATR FT-IR measurements were carried out using a Jasco FT-IR 4700 (MD, USA) instrument equipped with a diamond ATR attachment. The ATR FT-IR spectra were recorded in the range of 4000–400 cm^−1^ with a resolution of 4 cm^−1^ by taking 64 scans per each analysis. The collected data were then analyzed using Spectra Analysis software. In turn, UV/Vis absorption spectra were recorded using a Jasco V-530 (MD, USA) instrument in the range of 200–1100 nm with a resolution of 1 nm. Similarly, the gathered data were then investigated by applying Spectra Analysis software.

### Estimation of changes in the biological function of the pm-rf-APGD-treated DOX-containing solution

To establish the impact of the pm-rf-APGD treatment (under optimal operating parameters of the applied flow-through system) on the antibacterial properties of the DOX-containing solution, a standard disc-diffusion assay, involving the untreated and pm-rf-APGD-treated DOX solutions, was performed on bacterial strains classified as human opportunistic pathogens (Table 3). The selected strains are either model microorganisms frequently used in antibiotic-susceptibility testing or clinical isolates of pathogenic properties (Table 3). These strains originated from the collection of Intercollegiate Faculty of Biotechnology University of Gdansk and Medical University of Gdansk (IFB UG & MUG), in which they were stored at – 80 °C in 40% (v/v) glycerol. Before the experiments, bacterial cells (Table 3) were collected from a corresponding stock and spread in a reductive manner on a Mueller–Hinton Agar medium (M-HA; BTL, Poland). The inoculated plate was incubated at 37 °C for 24 h. Then, a loopful of the bacterial cells was collected from the M-HA plate and utilized for inoculation of 5 mL of the Mueller–Hinton broth (M-HB; BTL, Poland). Incubation of the culture for 24 h with 120 rpm shaking followed. Subsequently, the overnight culture was centrifuged (10 min; 6500 rpm). The supernatant was discarded and the precipitated bacterial cells were washed twice in sterile water. Optical density of the bacterial suspension was adjusted to 0.5 in the McFarland (McF) scale (approx. 1.5 × 10^8^ cells per mL) with the use of a DEN-1B (BioSan, Latvia) densitometer.

**Table 3:** Bacterial strains of opportunistic human pathogens used in this study.

Bacterial species	Strain nos	Cell morphology	Origin	Isolation	Other features	References
*Escherichia coli*	ATCC 25922 DSM 1103 CCUG 17620	Gram (−) rod	Clinical isolate from human	1946, USA	recommended reference strain for antibiotic susceptibility testing	^22^
*Staphylococcus aureus*	Newman NCTC 10833 ATCC 25904	Gram (+) cocci	Secondary infection of tubercular osteomyelitis in human	1952, UK	strong producer of coagulase	^23^
*Staphylococcus haemolyticus*	ATCC 29970 DSM 20263 NCTC 11042	Gram (+) cocci	Human skin	1975, USA	Type strain	^24^

ATCC—American Type Culture Collection (Manassas, USA); NCTC—National Collection of Type Cultures (Porton Down, UK); CCUG—Culture Collection University of Gothenburg (Gothenburg, Sweden); DSMZ—German Collection of Microorganisms and Cell Cultures (Brunswick, Germany).

The prepared 0.5 McF suspensions of bacterial cells (Table 3) were utilized for conducting the above-mentioned disc-diffusion assay. In this case, a sterile cotton swab was suspended in the prepared 0.5 McF bacterial suspension (Table 3). The excess liquid was discarded prior to spreading the collected bacterial suspension (Table 3) three times over the surface of the M-HA plate (20 cm^3^). Subsequently, blanc discs (Biomaxima, Poland) of 5 mm diameter were placed on the surface of the inoculated M-HA medium (3 discs per plate). 10 µl of either the pm-rf-APGD-treated DOX solution or the control untreated DOX-containing solution was poured on a blanc disc. Then, the M-HA plates were incubated at 4 °C for 1 h to allow for diffusion of the active substance into the medium. Next, the plates were subjected to 37 °C for 24 h prior to the measurement of the observed growth inhibition zones. This experiment was carried out in triplicate with three technical repeats each. The results were visualized and tested in terms of statistical significance with the use of R 3.1.3 ^25^. The observed diameters of the bacterial growth inhibition zones, resulting from subjection of the microbes to the pm-rf-APGD-treated DOX-containing solution, were compared with a two-sided t-test (either Student’s or Welch’s t-test depending on fulfillment of the requirements for the parametric analysis, as analyzed with Shapiro–Wilk and Levene’s tests) with the ones acquired for bacteria exposed to the untreated DOX-containing solution. *p* < 0.05 was implemented for performing all these calculations.

### Determination of RNS and ROS contents in the analyzed solutions

To evaluate the interactions and processes occurring at the interface of the pm-rf-APGD-system with the DOX solution, several colorimetric methods were employed. The concentrations of total ROS in addition to individual ROS and RNS, such as: H_2_O_2_, NO_2_^−^, NO_3_^−^, were assessed in the pm-rf-APGD-treated and untreated DOX solution under the optimal operating parameters established for this flow-through reaction-discharge system. The description of all methods used is presented in the Supplementary Information section. The determination of the RNS and ROS contents was performed 10 min after the pm-rf-APGD-treatment of the DOX-containing solution in order to cool it down to the room temperature.

### Assessment of major ROS, involved in the degradation process of DOX

To better assess the degradation pathway of DOX during the pm-rf-APGD treatment, the activities of significant ROS were estimated. In the degradation processes of DOX, the most important reactive constituents are ·OH, H_2_O_2_ and O_3_. The confirmation their involvements in DOX degradation process, the HPLC–DAD analyses for pm-rf-APGD-treated and untreated DOX solutions with addition of appropriate scavengers were carried out. The protocols for scavenging tests were described in details in the Supplementary Information.

## Results and discussion

### Development of the response surface regression models for the assessment of the impact of temperature and pH on decomposition of DOX in the pm-rf-APGD treated solutions

Regarding decomposition of antibiotics like DOX from aqueous solutions, little attention was paid so far to the possibility of using the systems generating CAPPs in direct contact with the liquids containing the dissolved antibiotic^16,20,26–28^. In all above-cited works, CAPPs were sustained in non-continuous flow systems, therefore, the treatment of the drug-containing solutions was of a non-flow character and considered rather limited volumes of these solutions, i.e. up to 50 mL maximum. Moreover, the treatment time applied in the above-reported CAPP systems was rather prolonged as it typically ranged from 2 to 80 min.

In the present work, a combined effect of different agents provided by pm-rf-APGD, including short-and long-term RONS, the UV radiation and other reactive species, including NO_x_, NH, N_2_, and N_2_^+^, produced in the gas phase of the discharge (see OES spectra presented in Fig. S2, Supplementary Information, for more details), was expected. All these agents might be responsible for efficient decomposition of DOX, being the tetracycline-class antibiotic. The construction of the proposed flow-through system allowed for a short contact of the solution with the plasma-associated components, hence, diminished the possibility of attributing the degradation of this organic compound to a too high temperature. In this way, the required effect was primarily linked with the plasma-liquid interactions, while the degradation process of the studied antibiotic was likely achieved via its interactions with the CAPP-derived reactive species. Considering the examined flow-through reaction-discharge system with pm-rf-APGD, it was expected that the most effective decay of DOX would be achieved at the highest solution temperature (but not > 42 °C as experimentally verified by using the duty cycles of 60% and higher) and the lowest pH of the CAPP-treated solution, indicating the formation of such short- and long-term RONS as HNO_2_ and HNO_3_, respectively^29^. For this reason, T and pH values of the DOX-containing solutions treated by pm-rf-APGD at different experimental conditions were the responses of the system in the Box-Behnken response surface design. After executing this experimental design according to the Box-Behnken matrix shown in Table S1, T of the pm-rf-APGD-treated DOX solution was established to range from 28.6 to 36.6 °C, while its pH changed from 2.94 to 3.28.

The established regression models for these two responses were statistically significant. As can be seen in Tables 1 and 2, the respective *p*-values for both models were equal to 0.009 (T) and 0.028 (pH), and lower than α = 0.1 used in the backward elimination of terms algorithm to find the final equations of these regression models. Corresponding R^2^ values were 66.9% and 63.1%, respectively. It was also worth of noting that that the *p*-values for the lack-of-fit test were much higher than the α value, i.e. 0.211 (T) and 0.430 (pH). This clearly indicated that the established equations adequately described the relationship (given in Tables 1 and 2) between the response variables and the studied operating parameters. Since there was no evidence that the developed regression models did not fit the acquired data, there was no reason to reject these models.

**Figure 1:**
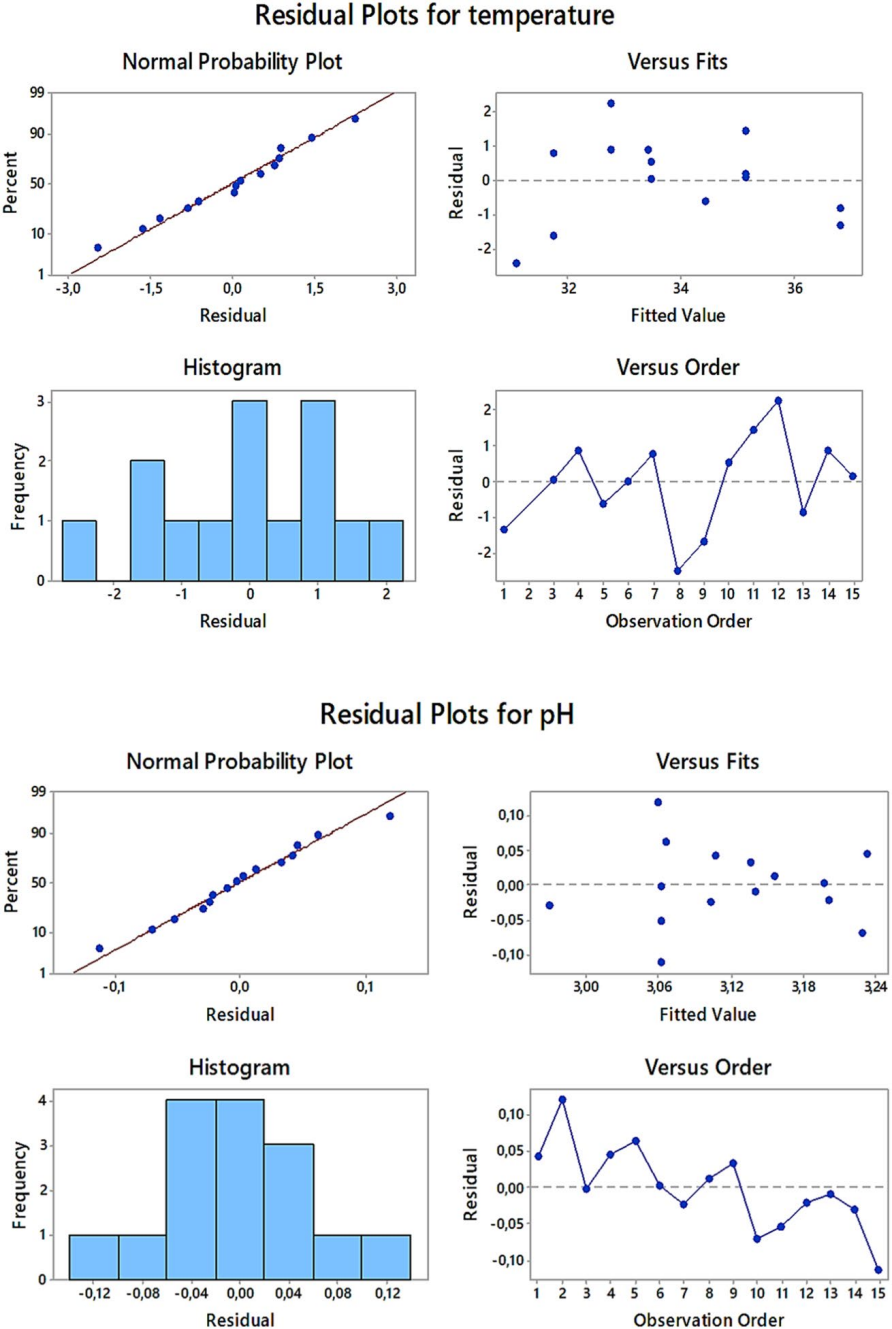
The residual plots for temperature (T) and pH, presenting the distribution of the analyzed residuals.

In addition, the residuals obtained for both regression models were visually analyzed (see Fig. 1). In the case of the normal probability plots, all data points followed the straight lines, confirming that they were normally distributed, both in the case of T and pH. When shifting to the scatter plots of the standardized residuals *versus* the run order, the data points were rather randomly placed on both sides of the center lines, proving that the residuals were not correlated between each other. All this pointed out that both regression models could be applied to properly select such operating parameters of the flow-through reaction-discharge system that would privilege efficient DOX decomposition.

### The effect of the operating conditions on temperature and pH of the DOX solution treated by pm-rf-APGD

**Figure 2:**
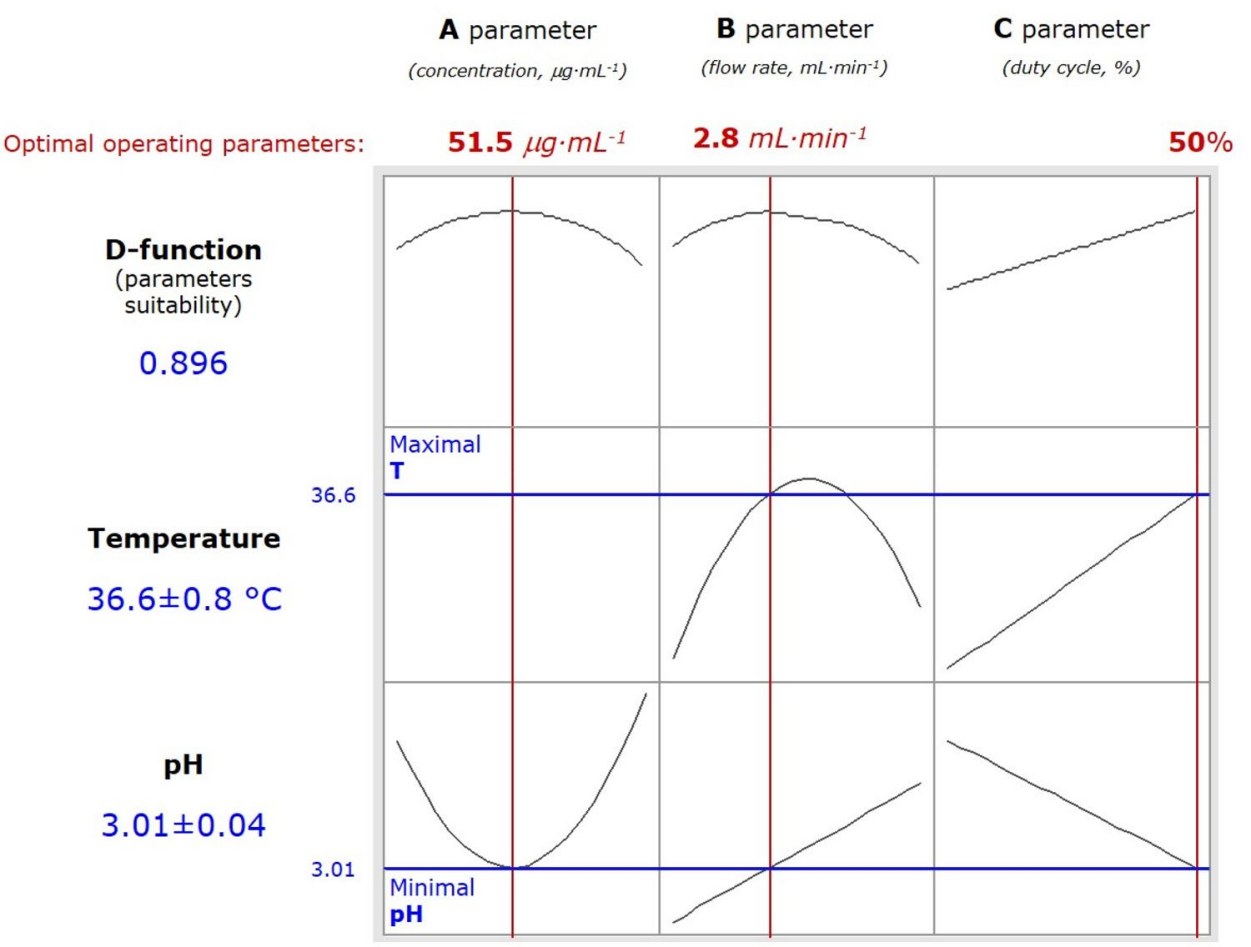
The effect of the pm-rf-APGD operating parameters on temperature (T, °C) and pH of the pm-rf-APGD-treated solution (according to the Box-Behnken matrix presented in Table S1).

Considering the effect of the operating parameters on T (°C) of the DOX solution treated by the flow-through pm-rf-APGD system, it seemed that it linearly increased while the duty cycle (the operating parameter C) of the pm-rf altering current supplying the discharge also elevated (Fig. 2). This could be directly related to the duration of impulses and the enhanced transfer of the electric field energy to the plasma, and consequently leading to intensification of the processes related to the plasma operation and the formation of ROS and RNS in the gas phase of the discharge and in the treated solutions. When referring to the effect of the FLE solution flow rate (the operating parameter B), it was established that T (°C) of such solution increased up to a certain value and then, it decreased, likely due to the cooling effect and a relatively short time of contact of the pm-rf-APGD plasma components with the solution consituents.

Taking into account pH of the DOX solution treated by pm-rf-APGD, the most favourable conditions, i.e. providing the lowest pH, were assured by the highest duty cycle (which coincided with its effect on T of the CAPP-treated solution) and the lowest flow rate of FLE solution. In the latter case, the contact time with the short- and long-term RONS in the gas phase of the discharge as well as the rate of plasma-liquid interactions would be the most intensive. This certainly would have an impact on the effective production of RONS in the solution, leading to efficient decomposition of DOX. The effect of the DOX concentration (the operating parameter A) was also found to be important to achieve a high degradation efficiency of DOX, following the pm-rf-APGD-treatment. Additionally, it was pointed out that the processed solutions and/or fluids containing DOX could eventually require to be initially diluted prior to the pm-rf-APGD treatment.

Considering both response surface regression models and the assumption of achieving an efficient antibiotic decomposition rate under the conditions of the highest T and the lowest pH (due to the processes of decomposition of the antibiotic by RONS generated via the plasma-liquid interactions), it was found that such conditions could be provided when the solution containing DOX at a concentration of 51.5 µg L^−1^ would be introduced at a flow rate of 2.8 mL min^−1^ to the pm-rf-APGD reaction-discharge system sustained by using the duty cycle of 50%. Under these conditions, the modeled response variables were established to be as follows: 36.6 ± 0.8 °C (T) and 3.01 ± 0.04 (pH). As such, the values of dT and dpH were 1.000 and 0.803, respectively. The value of the D function was 0.896, showing a reasonably good selection of the optimal conditions of the performed multiparameter optimization.

For the above-mentioned settings of the operating parameters (A: 51.5 µg mL^−1^, B: 2.8 mL min^−1^, C: 50%), both response surface regression models were validated. Independent experiments were carried out and the reaction-discharge system was run under the established operating parameters to treat a DOX solution. Then, T and pH of the pm-rf-APGD-treated solution were measured. The following results were obtained: T = 37.4 ± 0.1 °C and pH = 2.90 ± 0.10, and they were in a good agreement with the values predicted by the developed models.

### Determination of the efficiency of DOX degradation and identification of its degradation products, following pm-rf-APGD operation under the optimal operating conditions

The concentration of DOX in the untreated as well as pm-rf-APGD-treated solutions, prepared for the models validation, was determined using HPLC–DAD. It was found that in the prepared untreated DOX solution the quantified concentration of DOX was 54.5 µg mL^−1^ (instead of 51.5 µg mL^−1^). On the other hand, the concentration of DOX in the pm-rf-APGD-treated solution was 11.38 µg mL^−1^. Based on chromatograms presented in Fig. S3 (Supplementary Information), it was calculated that 79 ± 4.5% of DOX was effectively removed from the initial DOX solution by using the pm-rf-APGD treatment under the optimal operating parameters established for the applied flow-through reaction-discharge system.

**Figure 3:**
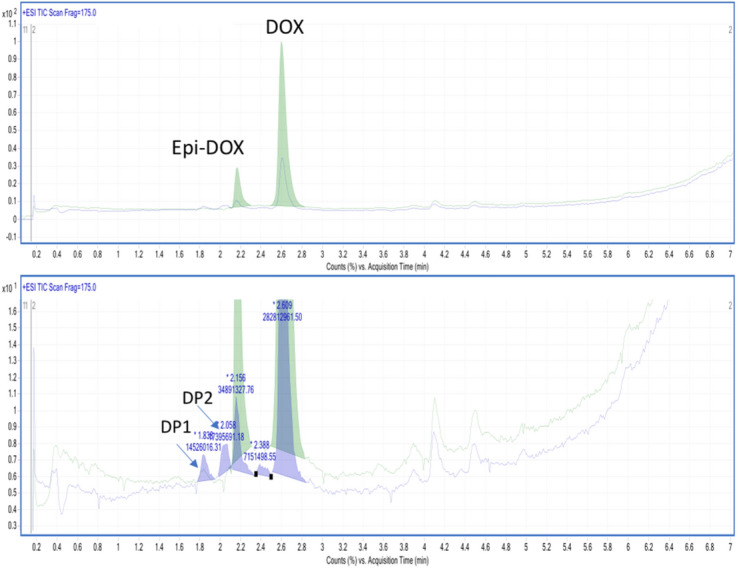
The upper part—the total ion current chromatogram (TIC) of DOX (the untreated solution); the lower part—the corresponding TIC with the added chromatogram of the pm-rf-APGD-treated DOX solution.

In the next step, it was crucial to identify the products of antibiotic degradation, because these substances might have an impact on the natural environment. For this reason, high resolution UPLC-MS/MS was used to identify the DOX degradation products, after treating the aqueous solution of this drug by pm-rf-APGD run under the optimal operating parameters. The total ion current chromatograms (TIC, ESI^+^) of the DOX solutions before and after the CAPP-treatment are presented in Fig. 3. On the chromatogram of the untreated DOX solution two peaks were detected with m/z of 445.16 [M + H]^+^. The peak at 2.6 min was from DOX, while the peak at 2.1 min most probably originated from epi-DOX, which is often found as an impurity of DOX and its abiotic transformation product. In the chromatogram of the pm-rf-APGD-treated DOX solution, two additional signals were found. The degradation product (DP) 1 gave the same mass spectra with [M + H]^+^ 461.1541. For this compound the software attributed the formula C_22_H_24_N_2_O_9_. This corresponds to DOX plus one O atom.

The MS/MS spectra (presented in Fig. S4, Supplementary Information) revealed that the fragmentation patterns of the main peak and the next one differed. The main fragmentation of DP1 occurred by a release of the OH group (m/z 17). The degradation product with m/z of 417.1649 corresponded to the formula C_21_H_24_N_2_O_7_ and could be assigned to DOX minus the CO group (Fig. S5, Supplementary Information). This mass also gave the second chromatographic peak, and the fragmentation pattern by MS/MS also gave [M-36 + H]^+^ (a release of the H_3_NO fragment) and [M-17 + H]^+^. All this suggested that during the pm-rf-APGD treatment of DOX such processes as the attack of the O radical and the removal of the CO from the chemical structure of DOX may be differentiated. Notably, in the chemical structure of DOX, there are several attract sides. In the final aqueous DOX sample, the concentration of these products was minor, which suggests that the mineralization of DOX was efficient.

After HPLC–DAD and UPLC-MS/MS analyses, the pm-rf-APGD-treated solution was subjected to the measurements of TOC and TN (changes in the overall content of C- and N-containing species/products in the solution) and the analysis by ATR FT-IR and UV/Vis (changes in the chemical structure of the target compound, group identification of new species/products present in the solution).

Firstly, the TOC and TN concentrations were determined in the untreated and pm-rf-APGD-treated DOX solutions to state the impact of pm-rf-APGD on the mineralization of the target antibiotic in the solution. It was found that after the pm-rf-APGD treatment of the DOX solution the TOC content slightly increased (***p < 0.004) from 32.28 ± 0.04 µg mL^−1^ (the untreated DOX solution) to 34.94 ± 0.42 µg mL^−1^ (the pm-rf-APGD-treated DOX solution). These changes might be associated with the slight water evaporation during the CAPP treatment of the DOX solution. Additionally, as was reported by Sarangapani et al.^16^, the observed deviations could be related to the formation of carboxylic acids in the CAPP-treated solution. Their presence might impact the effectiveness of the mineralization process of DOX by slowing it down. On the other hand, taking into consideration the changes in the TN concentration in the analyzed DOX solutions, it was assessed that the controlled pm-rf-APGD operation led to increase (****p < 0.0001) in the TN content by approximately 7.3 times; 23.81 ± 0.32 µg mL^−1^ in the untreated DOX solution vs. 3.25 ± 0.03 µg mL^−1^ in the pm-rf-APGD-treated DOX solution. This significant increase was likely caused by the production of HNO_3_ or HNO_2_ according to the following reactions: NO_2_ + OH = HNO_3_ and NO + OH = HNO_2_^30^. Additionally, the NO_2_^−^ ions were likely oxidated under the acidic conditions to the NO_3_^−^ ions according to the following reaction: 3HNO_2_ = NO_3_^−^  + 2NO + H_3_O^+^, as was previously reported by Jamróz et al.^30^ in terms of a dc-APGD system. The other paths for the production of HNO_3_ and HNO_2_ involved various forms of nitric oxide (NO_x_) with water and hydrogen peroxide: 2NO_2_ + H_2_O = NO_2_^−^  + NO_3_^−^  + 2H^+^, NO + NO_2_ + H_2_O = 2NO_2_^−^  + 2H^+^ . Moreover, the NO_2_^−^ ions are easily oxidized to NO_3_^–^ (e.g. NO_2_^−^  + H_2_O_2_ = NO_3_^−^  + H_2_O, NO_2_^−^  + O_3_ = NO_3_^−^  + O_2_, 3HNO_2_ = NO_3_^−^  + 2NO + H_3_O) or decomposed (2HNO_2_ = NO + NO_2_ + H_2_O) and thus, the concentration of these ions was relatively low^31^. The plasma-triggered production of HNO_3_ and HNO_2_ was confirmed by the measurements of pH of the untreated and pm-rf-APGD-treated DOX solutions. pH of the pm-rf-APGD-treated solution was an indicator of the RNS generation. The lowest pH was related to the enhanced production of the NO radicals (NO·), which was coincidently observed at the emission spectra of the pm-rf-APGD system. The NO radicals and their derivatives formed in the solution resulted in the formation of HNO_3_ (hence pH was lowered). Additionally, the highest production of ROS is observed, especially ·OH was observed in the lowest pH ^17^. Intensified production of ROS and RNS was responsible for the efficient degradation of the targeted compound. The goodness of this assumption was verified by the HPLC–DAD analyses of the pm-rf-APGD-treated solution under the optimal operating conditions (to the lowest pH and the highest T). In this case the efficiency of the degradation of the target compound was almost quantitative. By these means it was proven that measuring pH and T is a good way for optimizing the working parameters of the pm-rf-APGD system. For the here-analyzed solution, a drop in the pH value from 4.11 to 2.90 was detected, which confirmed the acidification of the DOX solution after the pm-rf-APGD treatment.

Then, ATR FT-IR and UV/Vis analyses of DOX solutions were conducted to define changes in the chemical structure of the target antibiotic after the pm-rf-APGD treatment. Based on the ATR FT-IR studies, no significant deviations in the conformation of DOX functionalities were identified. Irrespectively of either, the concentration of DOX in the solution or whether the solution before or after the pm-rf-APGD treatment was analysed, the presence of the bands associated with C = C (~ 1644 cm^−1^), and O–H (~ 3321 cm^−1^) bonds, as well as stretching vibrations attributed to the O–H and N–H (~ 2363 cm^−1^) bonds (Figure S6A)^32^ was confirmed. This could be related to the strong stretching vibrations attributed to the O–H bond, which possibly hindered the visualization of any changes in the DOX.

Moving to the UV/Vis analysis, there was a deviation observed in the spectra of the DOX solution obtained after the pm-rf-APGD treatment. As can be seen in Figure S6B, the pm-rf-APGD treatment of the DOX solution triggered a clear decrease in the absorbance value with simultaneous red-shifts of bands in the regions of ~ 360 and ~ 280 nm. The decreased absorbance could be linked with a dropped concentration of DOX following the pm-rf-APGD treatment and a dropped pH value of the solution. However, the above-mentioned changes could also be putatively associated with deviations in the chemical structure of DOX. This assumption coincident with the results obtained by using UPLC-MS/MS (Fig. 3).

To evaluate whether the pm-rf-APGD treatment leads to a decrease in the antibacterial properties of the DOX solution, the standard disc diffusion assay involving three strains of bacterial opportunistic human pathogens, i.e. *E. coli* ATCC 25922, *S. aureus* ATCC 25904 and *S. haemolyticus* ATCC 29970, was carried out.

**Figure 4:**
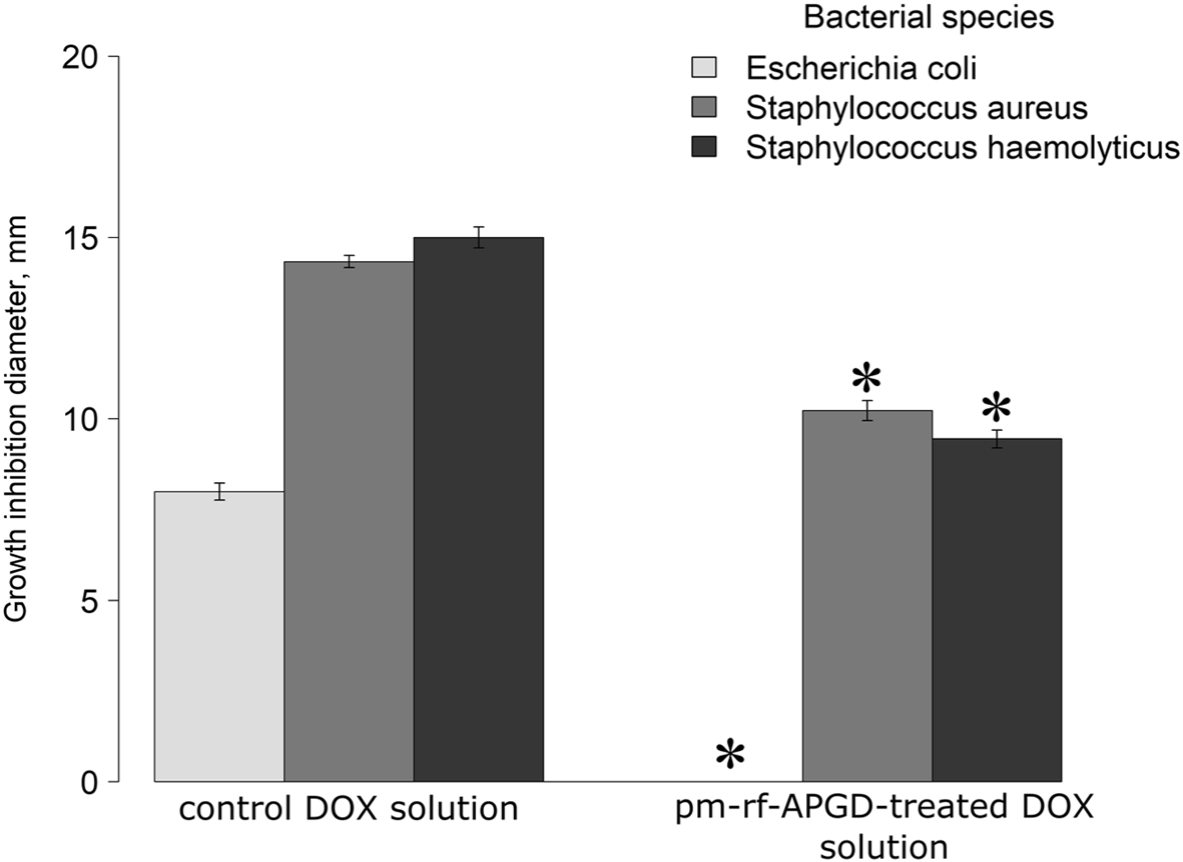
Antibacterial properties of the pm-rf-APGD-treated DOX solution in comparison to the non-plasma-treated control solution. A standard disc-diffusion assay was performed. Means ± standard errors of the measured bacterial growth inhibition diameters are depicted. Asterisks mark statistically significant differences (Welch Two Sample *t*-test at *p* < 0.05) between the antimicrobial actions of the CAPP-treated DOX solutions in contrast to the corresponding controls. The experiment was conducted three times with three technical repeats in each. The following bacterial strains were used: *E. coli* ATCC 25922, *S. aureus* ATCC 25904, and *S. haemolyticus* ATCC 29970. DOX – doxycycline. pm-rf-APGD – pulse-modulated radio-frequency atmospheric pressure glow discharge.

In terms of *E. coli* ATCC 25922, being a recommended reference strain for antibiotic susceptibility estimation, a complete loss of the antibacterial properties of the pm-rf-APGD-treated DOX solution was observed (Fig. 4). Concerning two other microorganisms included, i.e. clinical isolates *S. haemolyticus* ATCC 29970 and *S. aureus* ATCC 25904, statistically significant (Welch *t*-test; *p* < 0.05; Fig. 4) differences in the measured bacterial growth inhibition zones were recorded if the pm-rf-APGD-treated DOX solution was applied in contrast to the untreated DOX control solution. Decreases between the means of the growth inhibition diameters equalled 37% and 29%, concerning the assays conducted on *S. haemolyticus* ATCC 29970 and *S. aureus* ATCC 25904, respectively (Fig. 4).

Unfortunately, in the former studies applying different CAPP sources for degradation of DOX^20,21^, the crucial in our opinion issue regarding either loss or retaining the antimicrobial properties of the CAPP-treated DOX solutions has not been addressed. Previously, Zhang et al.^33^, reported the loss of the antimicrobial properties of a cefixime solution towards *E. coli* ATCC 25922 post the CAPP treatment inside bubbles of enlarged gas–liquid interfacial areas. This outcome was in accordance with our observation concerning the lack of the antibacterial action towards the same bacterial strain of the pm-rf-APGD-treated DOX solution. Similarly to the herein described data, Sarangapani et al.^16^, also observed a decrease in the antimicrobial action towards *E. coli* or *B. subtilis* strains of ciprofloxacin and ofloxacin suspended either in water or meat effluent post 15–25 min of a CAPP exposure. Therefore, we found indications that the plasma-based technology could be a solution to the frequently reported issue of ineffectiveness of the antibiotics removal by conventional wastewater management practices^34^.

Moreover, the studies of Li et al.^35^ and Liao et al.^36^ suggest further benefits of CAPP-based approaches for decontamination of liquid wastes from antibiotics. It seems that beside direct degradation of the molecules of biocidal activities, application of CAPP-based technology can also lead to elimination of multidrug resistant bacterial strains, degradation of DNA containing genes allowing acquisition of antibiotic resistance (e.g. *tet*(C), *tet*(W), *bla*TEM-1, *aac*(3)-II), and integron gene *intI*1), and diminution of the frequency of a horizontal gene transfer by conjugation^35,36^. Such complex outcomes of the CAPP-based treatment would be highly beneficial for decontamination of wastewaters from hospitals and clinics. For instance, Nguyen et al.^37^ monitored the degradation efficacy of ofloxacin, ciprofloxacin, cefuroxime and amoxicillin of the CAPP-based wastewater treatment plant in one of the Vietnam hospitals. The achieved drug degradation rates were high, i.e. exceeding 99% for cefuroxime and ciprofloxacin and 72% for amoxicillin and ofloxacin, though, sadly, the authors did not study the variation in the amount of the detected coliforms prior and post the CAPP-treatment. This aspect would be interesting to address in the future especially in a view of high microbiological contamination rates of wastewaters of a hospital origin (1.5 × 10^5^ − 1.4 × 10^7^ CFU/100 mL; Nguyen et al.^37^). A possible drawback of a CAPP-involving approach was listed by Gilmore et al.^38^, who reported induction of a transient, dormant, metabolically inactive phenotype providing elevated tolerance to stress in the CAPP-exposed microbial population, leading to generation of persister or viable but non-cultivable (VBNC) cells. Therefore, a research direction aiming for combining direct antimicrobial and drug-degrading properties of CAPPs is highly supported, though the experiments planned for confirmation of its effectiveness should not be solely based on standard microbiological cultivation approaches, which overlook a notable environmental pool of antibiotic resistance genes, namely the VBNC cells.

### Revealing the pm-rf-APGD-liquid interactions, leading to DOX degradation and decrease in its biological activity

To reveal the pm-rf-APGD-liquid interactions, which are involved in the DOX degradation process, the total concentration of ROS and the concentration of selected RONS, including NO_3_^−^, NO_2_^−^, and H_2_O_2_, were determined in the DOX-containing solution.

**Figure 5:**
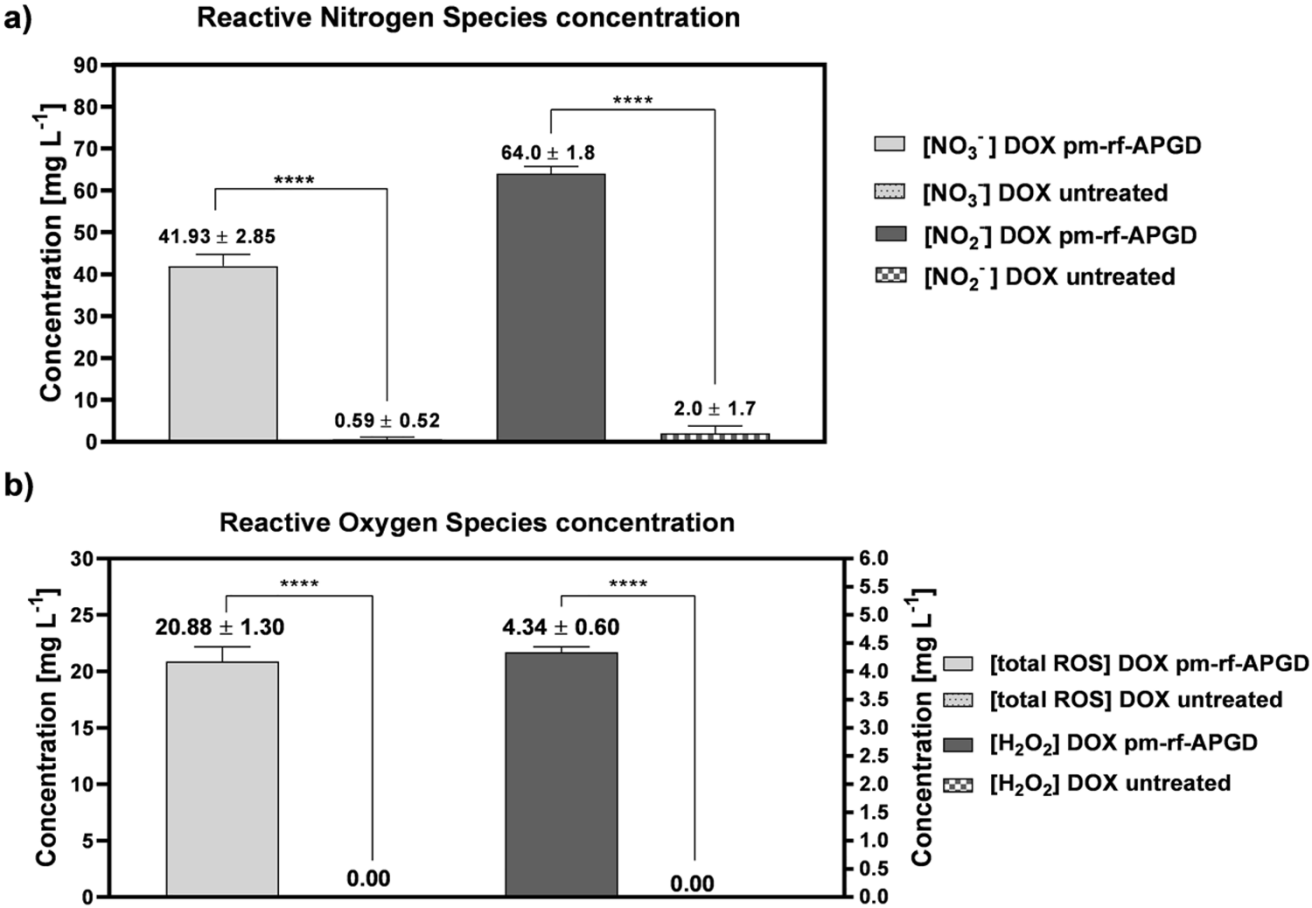
The concentration of reactive nitrogen (**a**) and oxygen (**b**) species determined in the pm-rf-APGD-treated DOX solution in addition to the untreated DOX solution. Means ± standard errors of the measured concentrations of the reactive forms are given. Asterisks mark: statistically significant differences (one-way analysis of variance with the Tukey’s post-hoc test; ****p < 0.0001).

It was found that the concentration of the measured RONS strongly increased after performing the pm-rf-APGD treatment of the DOX solution. Considering the total ROS concentration in the pm-rf-APGD-treated DOX solution, it reached 20.88 ± 1.30 µg L^−1^, while for the untreated DOX solution no ROS were determined (Fig. 5). Because H_2_O_2_ plays a key role in DOX degradation following the pm-rf-APGD treatment, the H_2_O_2_ concentration was also measured using other colorimetric assays. It was found that the concentration of H_2_O_2_ in the DOX solution, after the treatment by pm-rf-APGD, equalled 4.34 ± 0.60 mg L^−1^. For the untreated DOX solution, no H_2_O_2_ was detected. The determined H_2_O_2_ concentration is included in the estimated total amount of ROS. However, the contribution of H_2_O_2_ in the assessed total ROS concentration remains low, testifying to the significant involvement of the rest of ROS, especially ·OH and O_3_ in the DOX degradation.

The next step in further studies on the pm-rf-APGD-liquid interactions involved in the DOX degradation process was to determine the influence of lowered generation of the most crucial ROS on the degradation efficacy of DOX. By eliminating ·OH and separately H_2_O_2_ with O_3_ from the decomposition processes of DOX, it was possible to estimate the individual roles of these species in the occurring processes. It was found that the nucleophilic action of ·OH resulted in the 33.8% degradation efficacy as compared to the 83.5% degradation efficiency of DOX in the solution without ethanol. Additionally, the H_2_O_2_ scavenging in the pm-rf-APGD-treated DOX solution led to the 21.8% DOX degradation as compared to the above-mentioned 83.5% degradation efficiency of DOX in the solution without fetal bovine serum (FBS). Considering the obtained differences after scavenging the specific reactive individuals, it seemed that the decomposition of DOX was likely due to the presence of H_2_O_2_ and O_3_. From this point of view, the probable mechanism may occur through the action of the ·OH radical on the carbon–carbon double bond. The attachment of the subsequent species led to the formation of other intermediates, resulting in turn in decarboxylation or substitution of amino groups. All these phenomena can end up in the opening of the aromatic ring. For a complete overview of the possible intermediate products formed in these conditions and further reactions in which they take part, the paper by Magureanu et al.^17^ can be reffered to. On the other hand, the most significant elevations in the RNS content were observed in the case of the NO_3_^−^ ions, i.e. from 0.59 ± 0.52 mg L^−1^, determined in the untreated DOX solution, to 41.93 ± 2.85 mg L^−1^ measured in the pm-rf-APGD- treated DOX solution (Fig. 5). A similar pattern was observed in the case of the NO_2_^−^ ions, whose concentration increased after the pm-rf-APGD treatment of the DOX solution, i.e. from 2.0 ± 1.7 mg L^−1^ as quantified in the untreated DOX solution to 64.0 ± 1.8 mg L^−1^ determined in the pm-rf-APGD-treated DOX solution (Fig. 5). The significantly increased concentrations of the NO_2_^−^ and NO_3_^−^ ions confirmed generation of these RNS of a longer lifetime, following the pm-rf-APGD-liquid interactions. Furthermore, the additional source of mentioned RNS resulted from the decomposition of the DOX solution treated with the aid of the pm-rf-APGD system, confirming the effectiveness of this process. Moreover, the presence of the NO_2_^−^ and NO_3_^−^ ions in the liquid environment leads to the production of HNO_2_ and HNO_3_, sufficiently lowering the pH of the environment. The low acidity has a significant impact on the further cascade reactions, accelerating the generation of ·OH, which plays a crucial role in the degradation of DOX.

There is a broad spectrum of factors that has an impact on the properties of the CAPP treatment. Thus, the current–voltage characteristics of the discharge, the electrode configuration and the method of the solution introduction and its contact with the discharge significantly affect the contribution of major RNS and ROS to the drug decomposition process. In this perspective, utilization of dc-APGD-based reaction-discharge systems^39^, in which the solution of NH_4_NO_3_ acted as a flowing liquid cathode, resulted in production of a significantly lower, i.e. 9.3 µg mL^−1^, amount of the NO_2_^−^ ions than in the herein applied pm-rf-APGD system. There, the concentration of NO_3_^-^ ions in the untreated versus treated by the CAPP-based reaction-discharge system NH_4_NO_3_ solution was 1624 versus 1195 µg mL^−1^, respectively. On the other hand, the total ROS concentration was determined as 28.79 µg mL^−1^, which well agrees with the results presented in the present paper (20.88 ± 1.30 µg mL^−1^). However, considering the former analysis of the RONS concentration performed in more complex liquids such as culture media treated in a stationary regime by other CAPP sources, the determined concentrations of the studied RNS and ROS were comparable. In more detail, a dielectric barrier discharge (DBD), generated in contact with 1.50 mL of a DMEM culture medium led to the production of 10.00 ± 0.01 µg mL^−1^ of the NO_2_^−^ ions, 1.20 ± 0.08 µg mL^−1^ of the NO_3_^−^ ions, and 19.5 ± 0.4 µg mL^−1^ of the NH_4_^+^ ions as well as 3.31 ± 0.03 µg mL^−1^ of H_2_O_2_ molecules^40^. Despite the significantly lower concentrations of NO_2_^−^ and NO_3_^−^ ions recorded in the culture medium treated with CAPP, the concentrations of the NH_4_^+^ ions and H_2_O_2_ well corresponded to the values measured for these species in this paper. The concentrations of RONS were also measured in other plasma-treated buffers before. For instance, a CAPP treatment of 1.0 mL of PBS for 20 s led to the production of 1.7 µg mL^−1^ of H_2_O_2_^40^. In another study, activation of 2.0 mL of PBS for 5 min resulted in production of 17.72 µg mL^−1^ of the NO_2_^−^ ions and 1.28 µg mL^−1^ of the NO_3_^−^ ions, respectively^42^. Therefore, the herein reported plasma-liquid interactions yielded the production of the NO_2_^−^ and NO_3_^−^ ions, and H_2_O_2_ molecules, whose concentrations seemed to be in a good agreement with the concentrations reported in other studies.

As was described above, decomposition of DOX from the aqueous solution with the pm-rf-APGD system likely resulted from generation of RNS and ROS such as NO_3_^−^, NO_2_^−^, NH, N_2_, N_2_^+^, ·OH, and H_2_O_2_, as determined by OES in the gas phase of the CAPP system and the utilized colorimetric methods in the treated solution. As suggested in former studies, these reactive individuals exhibited high potency in elimination of chemical and biological contaminants from liquid waste^43^. Here, we demonstrated that beside 79 ± 4.5% degradation of DOX by RONS produced in the utilized pm-rf-APGD system, the CAPP-treated DOX solution either completely lost its biocidal activity or had it significantly decreased, depending on the investigated bacterial species.

In this view, we expect a diminishment in the selective pressure posed by the pm-rf-APGD-treated solutions and, thus, we could assume also lower rates of horizontal gene transfer and a limitation in the selection of free-living microbial populations with genes determining antibiotic resistance^7,8^. As a result, these genetic determinants will not reach so effectively human pathogenic bacteria of high clinical importance. Therefore, antibiotics, including in our case DOX, will still remain an available therapeutic option. Our research has proven that pm-rf-APGD-based plasma might be an efficient, innovative and cost-effective alternative for the treatment of wastewater contaminants, including the ones difficult to cope with^44^. Although, having in mind possible future applications of the herein reported pm-rf-APGD-based reaction-discharge system for wastewater purification purposes, it must be assured that the CAPP-treated liquids will not pose any environmental risks and the generated disposals will not disrupt sustainable ecological processes in the biosphere^45^. One of the aspects to be addressed would be how long the generated RONS exhibit their red-ox potential. Also rather low pH of the pm-rf-APGD-treated solution should be neutralized, e.g. during denitrification processes in the conventional wastewater facilities^44^, or with the use of lime or cement kiln dust^46^. Furthermore, during a future scale-up and adjustment of this technology for industrial requirements, we need to take into consideration that a fraction of the pm-rf-APGD-generated RONS shall be consumed by the wastewater matrix^36^, therefore, leaving lower concentrations of these individuals accessible for the contaminants-degradation process. The above-listed issues shall be further investigated in our future research.

## Conclusions

We have shown for the first time that the developed and optimized flow-through pm-rf-APGD-based reaction-discharge system could be efficiently applied for decomposition of DOX from aqueous solutions and therefore limit the antimicrobial properties of this drug. To find the optimal operating parameters of the developed reaction-discharge system under which the DOX solution will be successfully degraded, its multivariate optimization was conducted, revealing the effects of the CAPP operating parameters on pH and temperature of the subjected to CAPP DOX solution. Based on the statistical analysis, it was found that the flow rate of the DOX solution, the duty cycle for the pulse-modulated radio-frequency alternating current, and the DOX concentration in solution play crucial roles in achieving the intended operating conditions, i.e. parameters under which T of the CAPP-treated solution will be the highest while the pH will be the lowest. By subjecting the DOX solution to pm-rf-APGD run under the established optimal operating parameters, the 79 ± 4.5% decomposition rate of DOX was noted. The degradation products of DOX were DOX plus one O atom and DOX minus CO. The changes in the DOX UV/Vis spectra were detected post the CAPP treatment, which further confirmed decomposition of this antibiotic to lower molecular weight products. The pm-rf-APGD-treated DOX solution exhibited no antimicrobial properties towards the model strain for antibiotic susceptibility testing, i.e. *E. coli* ATCC 25922, and significant decreases by 37% and 29% in its biocidal activities towards clinical isolates *S. haemolyticus* ATCC 29970 and *S. aureus* ATCC 25904, respectively. By revealing the CAPP-liquid interactions it was found that ROS and RNS, in our case NO_3_^−^, NO_2_^−^, NH, N_2_, N_2_^+^, OH, and H_2_O_2_, were involved in DOX decomposition. We foresee that future implementation of the herein developed efficient, innovative and cost-effective DOX degradation method into wastewater treatment facilities will provide benefits for the public health sector by lowering the frequency of the occurrence of multidrug-resistant strains. Therefore, the treatment of bacterial infections under hospital settings still could be handled with the use of commonly utilized antibiotics.

## Supplementary Information

Supplementary Information.

## Abbreviations

ACN: Acetonitrile
ANOVA: Analysis of variance
ATR FT-IR: Attenuated total reflectance Furrier Transform-Infrared
CAPP: Cold atmospheric pressure plasma
DoE: Design of experiment
DOX: Doxycycline
DP: Degradation product
FLE: Flowing liquid electrode
FVB: Full vertical biding
HPLC–DAD: High-performance liquid chromatography–diode array detection
LC-QToF MS: Liquid chromatography quadrupole time of flight mass spectrometry
LOD: Limit of detection
LOQ: Limit of quantification
M-HA: Mueller–Hinton Agar
M-HB: Mueller–Hinton Broth
NPOC: Non-purgeable organic carbon
OES: Optical emission spectrometry
pm-rf-APGD: Pulse-modulated radio-frequency atmospheric pressure glow discharge
RNS: Reactive nitrogen species
ROS: Reactive oxygen species
RONS: Reactive oxygen and nitrogen species
RSD: Relative standard deviation
RSM: Response surface methodology
TOC: Total organic carbon
TN: Total nitrogen
UPLC–MS/MS: Ultraperformance liquid chromatography–tandem mass spectrometry
UV/Vis: Ultraviolet–visible
VBNC: Viable but non-cultivable
