# Supplementary material for: Application of pulse-modulated radio-frequency atmospheric pressure glow discharge for degradation of doxycycline from a flowing liquid solution
Source: Sci Rep. 2022 May 5;12:7354. doi: 10.1038/s41598-022-11088-w (PMC9072311; doi:10.1038/s41598-022-11088-w)
Supplement: Supplementary file 1 — Supplementary Information. [file 41598_2022_11088_MOESM1_ESM.docx]

**SUPPLEMENTARY INFORMATION**

**Application of pulse-modulated radio-frequency atmospheric pressure glow discharge for degradation of doxycycline from a flowing liquid solution**

Anna Dzimitrowicz^1*^, Magda Caban^2^, Dominik Terefinko^1^, Pawel Pohl^1^, Piotr Jamroz^1^, Weronika Babinska^3^, Piotr Cyganowski^4^, Piotr Stepnowski^2^, Ewa Lojkowska^3^, Wojciech Sledz^3^, and Agata Motyka-Pomagruk^3^

^1^ Wroclaw University of Science and Technology, Department of Analytical Chemistry and Chemical Metallurgy, 27 Wybrzeze St. Wyspianskiego, 50-370 Wroclaw, Poland

^2^ University of Gdansk, Faculty of Chemistry, Department of Environmental Analysis, 63 Wita Stwosza, 80-308 Gdansk, Poland

^3^ University of Gdansk, Intercollegiate Faculty of Biotechnology University of Gdansk and Medical University of Gdansk, Laboratory of Plant Protection and Biotechnology, 58 Abrahama, 80-307 Gdansk, Poland

^4^ Wroclaw University of Science and Technology, Department of Polymer and Carbonaceous Materials, 27 Wybrzeze St. Wyspianskiego, 50-370 Wroclaw, Poland

*Corresponding Author: Dr. Anna Dzimitrowicz; Phone +48 71-320-24-53; e-mail address: anna.dzimitrowicz@pwr.edu.pl

**This Supplementary Information contains detailed Methodology dedicated to determination of the Reactive Oxygen Species (ROS) and Reactive Nitrogen Species (RNS) contents in the analyzed solutions and the assessment of major ROS, involved in the degradation process of doxycycline (DOX). Furthermore, the Figures S1-S6 and Table S1 have been included.**

**Figure S1 includes a graphical representation of the pm-rf-APGD-based reaction-discharge system. Figure S2 shows the OES spectrum of the cold atmospheric pressure plasma (CAPP) operation in contact with a flowing DOX solution. Figure S3 displays the HPLC-DAD chromatograms of untreated and pm-rf-APGD-treated DOX solutions. Finally, Figure S4 and Figure S5 show MS**/**MS mass spectra of the DOX degradation products. Figure S6 depicts the UV/Vis and ATR FT-IR spectra of untreated and treated by pm-rf-APGD DOX solution. Table S1 presents a BOX-Behnken design matrix with CAPP working conditions for DOX treatment.**

**Methods**

**Determination of the ROS and RNS contents in the analyzed solutions.** In order to assess the total concentration of ROS in addition to the concentration of selected ROS and RNS, including NO_2_^-^, NO_3_^-^, H_2_O_2_, several colorimetric methods were used as follows:

1. **Total ROS:** The total concentration of ROS, produced in the analyzed DOX solutions, was assessed by using a KI-starch based method (Mitsugi et al., 2021) ^1^. In this method, the reagent suspension, consisting of 0.3% (m/v) potassium iodide and 0.5% (m/v) starch, reacts with molecules whose oxidative potential exceeds 0.54 V, therefore turning the reaction mixture into blue color. The quantitative analysis of total ROS present in the CAPP-treated DOX solution was performed by measuring the absorbance of the above-mentioned reaction mixture at 590 nm with a Specord 210 Plus (Analytik Jena, Germany) instrument. The spectrophotometer was zeroed with de-ionized water. The experimentally acquired absorbance values were converted on the basis of a five-point calibration curve in the H_2_O_2_ concentration range of 1.75 -17.00 μg mL^-1^. The assay was carried out in three independent replicates. The results were expressed in μg mL^-1^ of H_2_O_2._
2. **H_2_O_2_ molecules:** The quantitative determination of the H_2_O_2_ concentration in DOX solutions, subjected to the pm-rf-APGD treatment or not, was carried out by using a colorimetric method based on the reaction with ammonium metavanadate (NH_4_VO_3_) (Nogueira et al., 2005) ^2.^ In this method, the reagent solution, consisting of 6.2 mmol L^-1^ of NH_4_VO_3_ and 5.8 mmol L^-1^ of H_2_SO_4_, reacted with H_2_O_2_, leading to the formation of a color product, whose λ_max_ was at 450 nm. The absorbance of the resulting reaction mixture at 450 nm was measured by using a Specord 210 Plus (Analytik Jena, Germany) instrument. The experimentally acquired absorbance values were converted on the basis of a five-point calibration curve in the H_2_O_2_ concentration range of 1.75-17.00 μg mL^-1^. The spectrophotometer was zeroed with de-ionized water. The assay was performed in three independent replicates.
3. **NO_2_^-^ ions:** A HANNA HI 96707 spectrophotometric kit (HANNA Instruments, Poland) was used to assess the NO_2_^-^ ions content in the pm-rf-APGD-treated DOX solution. The measurements were conducted following the instructions provided by the manufacturer. As a control, the untreated DOX solution was used. The measurements were performed in three independent repetitions. The results were the mean values expressed in μg mL^-1^.
4. **NO_3_^-^ ions:** A HANNA HI 96728 spectrophotometric kit (HANNA Instruments, Poland) was applied to assess the NO_3_^-^ ions content in the pm-rf-APGD-treated DOX solution. The measurements were conducted according to the instructions provided by the manufacturer. As a control, an untreated DOX solution was used. The measurements were performed in three independent repetitions. The results were the mean values expressed in μg mL^-1^.

**Assessment of major ROS, involved in the degradation process of DOX.** To elucidate the degradation mechanism of DOX, the addition of the radicals scavenger was conducted in order to lower the impact of major ROS, such as H_2_O_2_, O_3_ as well as •OH radicals, to the DOX degradation process. The scavenger analyses were performed as follows:

1. **Scavenging of H_2_O_2_**: In the experiment aiming for diminishment of the H_2_O_2_ content during DOX degradation, Fetal Bovine Serum (FBS) was used as the H_2_O_2_ and O_3_ scavenger. A 10% FBS solution was added to all of the studied DOX solutions before the plasma treatment and then after the treatment by pm-rf-APGD under the optimal working conditions. The collected post-treatment solutions were analyzed in terms of the DOX degradation efficacy using the HPLC-DAD technique.
2. **Scavenging of •OH**: ethanol was used to assess the •OH involvement in the CAPP-mediated degradation process of DOX. In more details, ethanol was introduced into DOX solutions before the pm-rf-APGD treatment, establishing the molar ratio of ethanol to DOX 1000:1. Then, the solutions were treated by pm-rf-APGD under optimal working conditions. Afterwards, the post-treatment DOX solutions were collected and analyzed using the HPLC-DAD technique. The obtained degradation efficacies were compared to these recorded for DOX solutions without addition of the scavenger agent. This approach enabled to establish the contribution of this radical to the DOX degradation process.


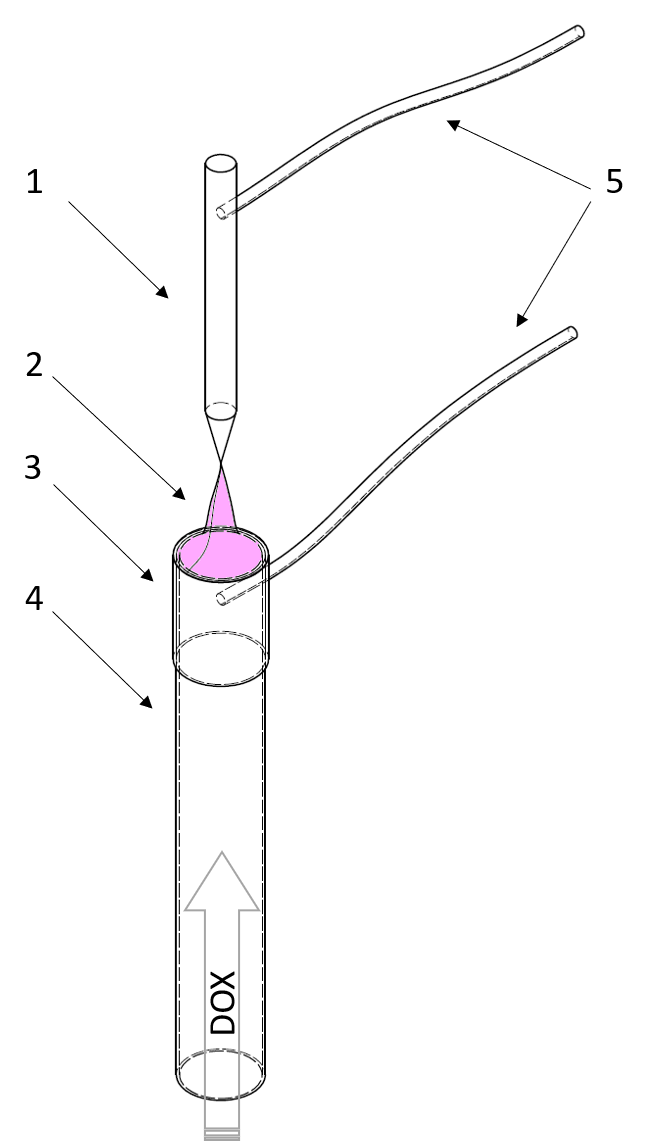


**Figure S1**. A graphical representation of the pm-rf-APGD-based reaction-discharge system for the flow-through treatment of the DOX-containing solutions. (1) sharpened tungsten electrode, (2) pm-rf-APGD, (3) graphite electrode, (4) quartz capillary, (5) high voltage inputs. The DOX solution is continuously delivered into the system *via* the graphite tube (3) mounted onto the quartz capillary (4). pm-rf-APGD (2) is operated in the gap between the metallic sharpened electrode (1) and the surface of the DOX solution.


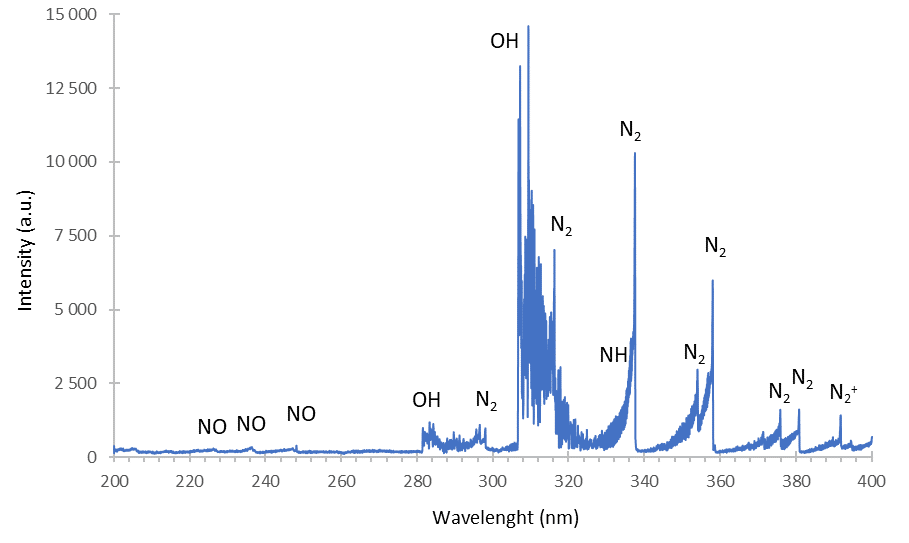


**Figure S2.** The emission spectrum of the pm-rf-APGD-based reaction-discharge system measured in the UV range (200-400 nm).

The emission spectrum of pm-rf-APGD, operated in the developed flow-through reaction-discharge system in contact with a flowing DOX solution and under the optimal operating parameters, is presented in Figure S1. The spectrum was dominated by the emission bands of the N_2_ (C-B) system with the band heads at 276.2 nm (2-0), 315.9 nm (1-0), 337.1 nm (0-0), 353.7 nm (1-2) 357.7 nm (0-1), 375.5 (1-3), and 380.4 nm (0-2). Additionally, numerous bands of the γ-system of NO (A-X) were noted in the range of 200-260 nm. The most intense band heads of this NO system were identified at 226.2 nm (0-0), 236.3 nm (0-1), and 247.1 nm (0-2). The OH (A-X) system was also excited (two band heads at 282.9 nm and 308.9 nm). Moreover, the (0-0) band head of the NH (A-X) system at 336.0 nm and the (0-0) band head of the N_2_^+^ (B-X) system at 391.4.nm were also identified. In the range above 400 nm, the H atomic lines at 486.1 nm and 656.2 nm as well as the O atomic lines at 777.2 nm, 777.4 nm, and 844.6 nm were observed.


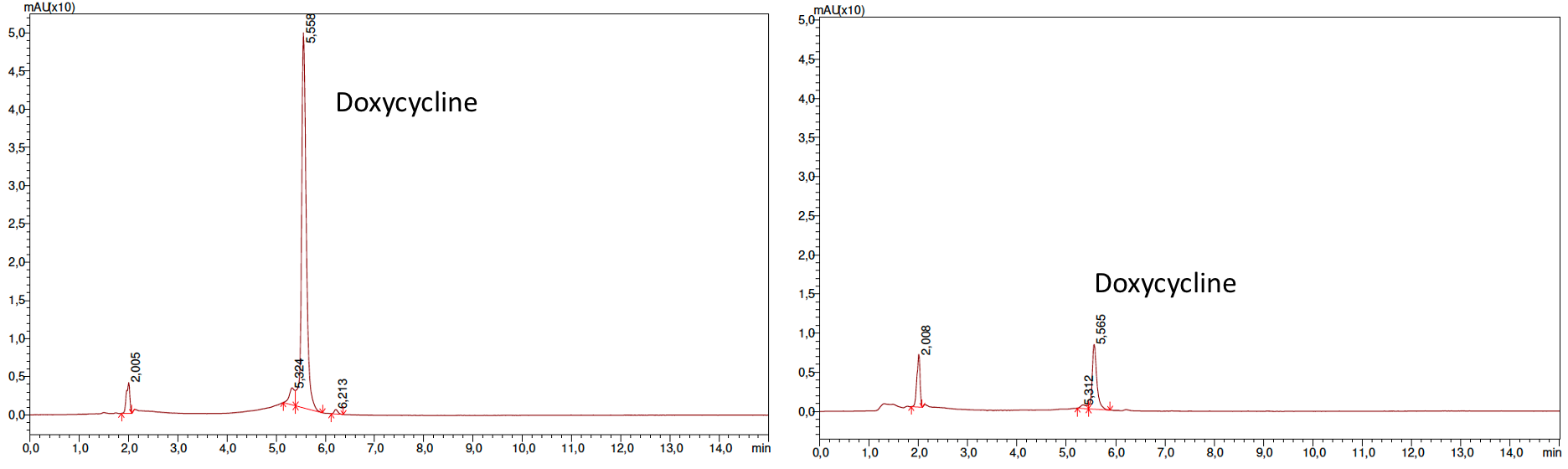


**Figure S3.** The HPLC-DAD (348 nm) chromatograms of (left) the DOX solution before the pm-rf-APGD treatment and (right) the DOX solution after the pm-rf-APGD treatment.


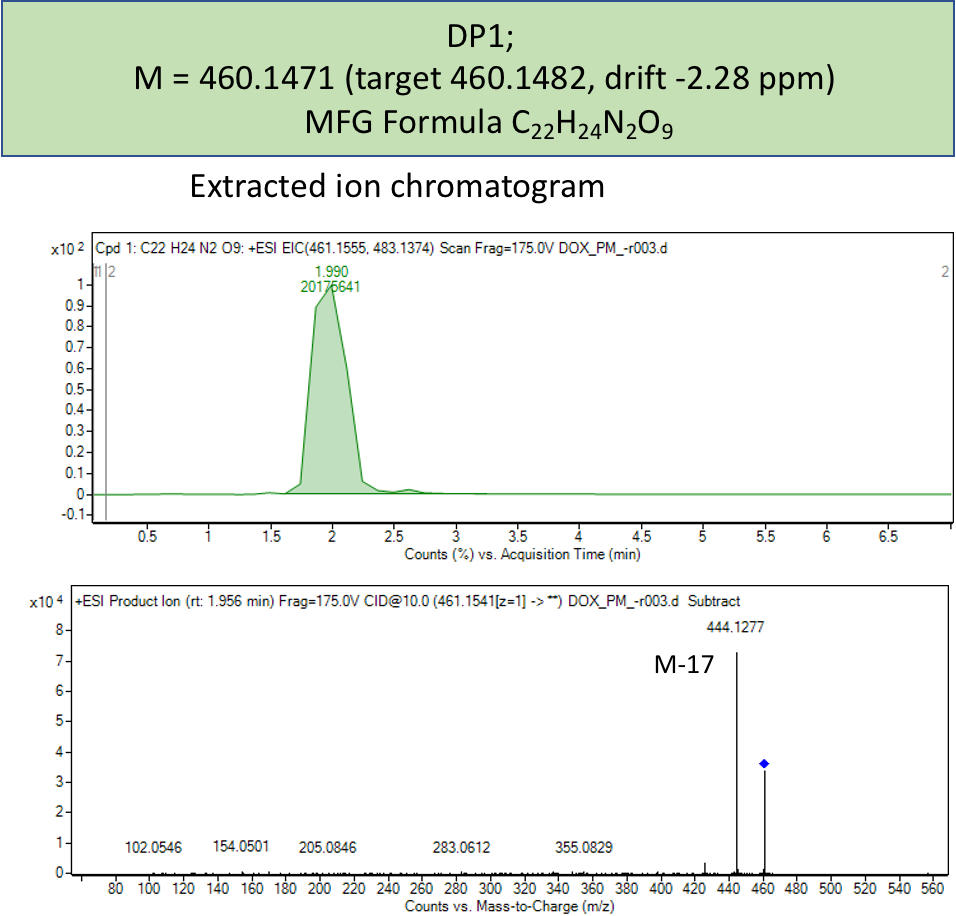


**Figure S4.** The MS/MS mass spectrum of the DOX degradation product with [M+H]^+^ 461.154 m/z.


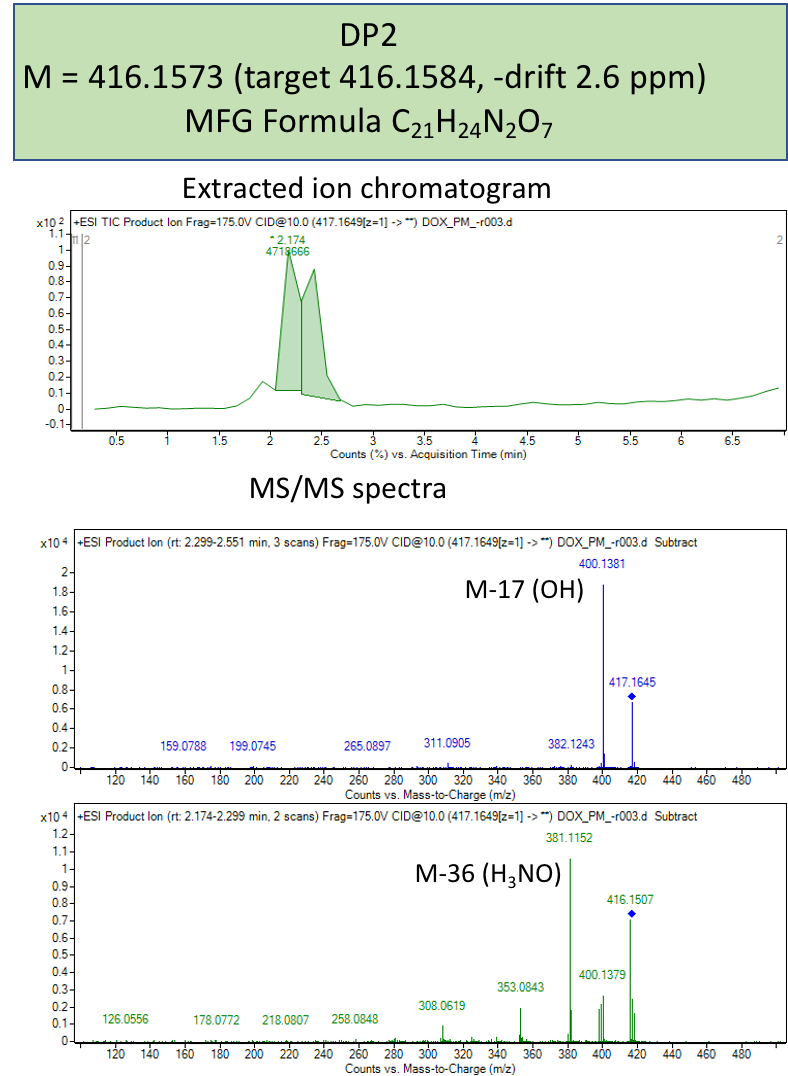


**Figure S5.** The MS/MS mass spectra of the DOX degradation product with [M+H]^+^ 417.1649 m/z.


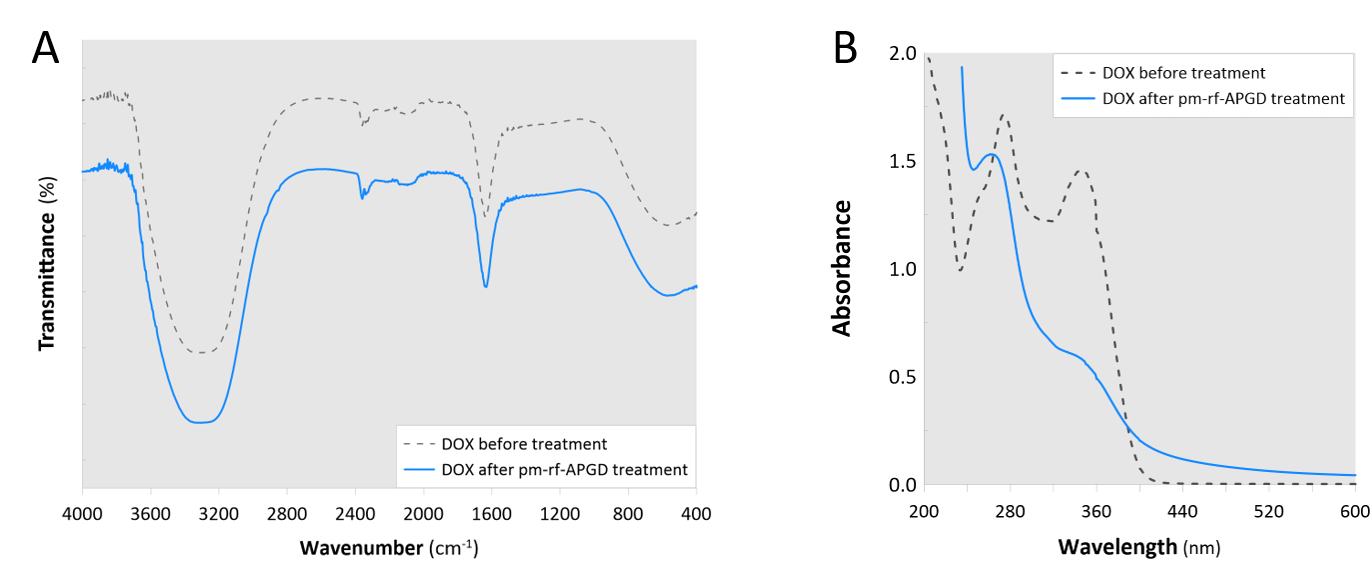


**Figure S6**. (A) ATR FT-IR, and (B) UV/Vis spectra of the untreated and pm-rf-APGD-treated DOX solution.

**Table S1.** The Box-Behnken response surface design matrix applied for performing a multiparameter optimization of the DOX decomposition process with the flow-through pm-rf-APGD-based system. The settings of the studied operating parameters, *i.e.* A: the DOX concentration, B: the FLE solution flow rate, C: the duty cycle for the pulsed radio-frequency alternating current, and the run order are given. The responses of the pm-rf-APGD system were temperature (T, **°**C) and pH of pm-rf-APGD-treated solutions.

| **Standard order** | **Run order** | **Number of blocks** | **A,**  **μg mL^-1^** | **B, mL min^-1^** | **C, %** | **T** (°C) | **pH** |
| --- | --- | --- | --- | --- | --- | --- | --- |
| 7 | 1 | 1 | 10 (-1) | 3 (0) | 50 (+1) | 35.5 | 3.15 |
| 9 | 2 | 1 | 55 (0) | 2 (-1) | 30 (-1) | 35.3 | 3.18 |
| 13^a^ | 3 | 1 | 55 (0) | 3 (0) | 40 (0) | 35.2 | 3.06 |
| 4 | 4 | 1 | 100 (+1) | 4 (+1) | 40 (0) | 33.6 | 3.28 |
| 12 | 5 | 1 | 55 (0) | 4 (+1) | 50 (+1) | 33.8 | 3.13 |
| 5 | 6 | 1 | 10 (-1) | 3 (0) | 30 (-1) | 33.5 | 3.20 |
| 1 | 7 | 1 | 10 (-1) | 2 (-1) | 40 (0) | 32.5 | 3.08 |
| 10 | 8 | 1 | 55 (0) | 4 (+1) | 30 (-1) | 28.6 | 3.17 |
| 2 | 9 | 1 | 100 (+1) | 2 (-1) | 40 (0) | 30.1 | 3.17 |
| 6 | 10 | 1 | 100 (+1) | 3 (0) | 30 (-1) | 34.0 | 3.16 |
| 15^a^ | 11 | 1 | 55 (0) | 3 (0) | 40 (0) | 36.6 | 3.01 |
| 3 | 12 | 1 | 10 (-1) | 4 (+1) | 40 (0) | 35.0 | 3.18 |
| 8 | 13 | 1 | 100 (+1) | 3 (0) | 50 (+1) | 36.0 | 3.13 |
| 11 | 14 | 1 | 55 (0) | 2 (-1) | 50 (+1) | 34.3 | 2.94 |
| 14^a^ | 15 | 1 | 55 (0) | 3 (0) | 40 (0) | 35.3 | 2.95 |

^a^ Center points.

**References**

1. Mitsugi, F., Kusumegi, S., Nishida, K. & Kawasaki, T. Visualization of Plasma-Induced Liquid Flow Using KI-Starch and PIV. *IEEE Trans. Plasma Sci.* **49**, 9–14 (2021).

2. Nogueira, R.F.P., Oliveira, M.C. & Paterlini, W.C. Simple and fast spectrophotometric determination of H_2_O_2_ in photo-Fenton reactions using metavanadate. *Talanta* **66**, 86–91 (2005).
